# Organic Residue Amendments to Modulate Greenhouse Gas Emissions From Agricultural Soils

**DOI:** 10.3389/fmicb.2018.03035

**Published:** 2018-12-07

**Authors:** Kristof Brenzinger, Sytske M. Drost, Gerard Korthals, Paul L. E. Bodelier

**Affiliations:** Department of Microbial Ecology Netherlands Institute of Ecology (NIOO-KNAW), Wageningen, Netherlands

**Keywords:** nitrous oxide, carbon dioxide, methane oxidation, agricultural soil, organic amendment, flux measurements, qPCR

## Abstract

Organic fertilizers have been shown to stimulate CH_4_ uptake from agricultural soils. Managing fertilizer application to maximize this effect and to minimize emission of other greenhouse gasses offers possibilities to increase sustainability of agriculture. To tackle this challenge, we incubated an agricultural soil with different organic amendments (compost, sewage sludge, digestate, cover crop residues mixture), either as single application or in a mixture and subjected it to different soil moisture concentrations using different amounts of organic amendments. GHG fluxes and *in vitro* CH_4_ oxidation rates were measured repeatedly, while changes in organic matter and abundance of GHG relevant microbial groups (nitrifiers, denitrifiers, methanotrophs, methanogens) were measured at the end of the incubation. Overall the dynamics of the analyzed GHGs differed significantly. While CO_2_ and N_2_O differed considerably between the treatments, CH_4_ fluxes remained stable. In contrast, *in vitro* CH_4_ oxidation showed a clear increase for all amendments over time. CO_2_ fluxes were mostly dependent on the amount of organic residue that was used, while N_2_O fluxes were affected more by soil moisture. Several combinations of amendments led to reductions of CO_2_, CH_4_, and/or N_2_O emissions compared to un-amended soil. Most optimal GHG balance was obtained by compost amendments, which resulted in a similar overall GHG balance as compared to the un-amended soil. However, compost is not very nutrient rich potentially leading to lower crop yield when applied as single fertilizer. Hence, the combination of compost with one of the more nutrient rich organic amendments (sewage sludge, digestate) provides a trade-off between maintaining crop yield and minimizing GHG emissions. Additionally, we could observe a strong increase in microbial communities involved in GHG consumption in all amendments, with the strongest increase associated with cover crop residue mixtures. Future research should focus on the interrelation of plants, soil, and microbes and their impact on the global warming potential in relation to applied organic amendments.

## Introduction

The atmospheric concentrations of the main GHG carbon dioxide (CO_2_), methane (CH_4_), and nitrous oxide (N_2_O) increased dramatically since the industrial revolution by 40, 150, and 20%, respectively (Stocker et al., 2013). Primarily, anthropogenic activities have increased the emission of CO_2_, CH_4_, and N_2_O. An estimated part of ~50% for CH_4_ and ~60% for N_2_O originates from agricultural practices (Tian et al., 2016). Intensification of agricultural land used to meet the global food, feed, and bioenergy demand for the growing human population entails increasing reinvestment of climate neutral carbon compounds (residues) into agricultural systems to prevent decline of soil organic matter and subsequent soil quality and fertility. However, agricultural intensification through increased fertilization can lead to the loss of soil CH_4_ uptake capacity (Bodelier and Steenbergh, 2014) and additionally causes an enhanced emission of N_2_O by lowering the reduction of N_2_O to N_2_. Particularly, CH_4_ uptake was 3–9 times weaker in agricultural than in unmanaged soils (Maxfield et al., 2008; Levine et al., 2011; Tate, 2015). Two major groups of fertilizers can be distinguished: organic fertilizer (e.g., compost, manure) and mineral forms (e.g., extracted from minerals or produced industrially) which both have been shown to strongly affect GHG emissions (Hallin et al., 2009; Syakila and Kroeze, 2011; Thangarajan et al., 2013; Shaaban et al., 2016). A common problem of mineral fertilizers is the loss of N and P by leaching (Kramer et al., 2006) and the decreased soil pH by repeated addition of N-fertilizer (Cheng et al., 2015) which by itself can give rise to enhanced N_2_O emissions (Bakken et al., 2012). Organic amendments represent a more sustainable fertilization strategy as they convey more efficient retention of nitrogen and carbon compounds necessary for plant growth. These organic amendments, like composted cattle manure, biochar, or zeolite addition or crop residue addition can also lower the emission of N_2_O, or increase its reduction to N_2_ (Thomson et al., 2012; Thangarajan et al., 2013). However, regarding the GHG related, underlying microbiology under influence of fertilizer applications, knowledge is far from complete.

Recent novel insights led to the postulation that representatives of the newly discovered N_2_O-reducing clade II can possibly turn soils into sinks of N_2_O (Jones et al., 2014; Domeignoz-Horta et al., 2015). However, attempts to stimulate soil N_2_O uptake by inoculation with a non-denitrifying *nosZ* clade II strain lowered the net potential emission but did not turn the soil into a sink of N_2_O (Domeignoz-Horta et al., 2016b). While the soil sink function of N_2_O still has to be verified, CH_4_ uptake can be found in several soils thereby contributing to cooling side of the GHG balance, representing 6% of the total global methane sink (Kirschke et al., 2013; Tian et al., 2016). However, fertilizer effects on the CH_4_ sink function in agricultural soils have received far less attention as compared to wetlands and well-aerated non-agricultural soils. This is due the very low or negligible methane uptake capacity in these soils as compared to grassland and upland forest soils (Mosier and Delgado, 1997; Veldkamp et al., 2013; Ciais et al., 2014). By converting natural soils into agricultural soils, up to a 7-fold reduction of CH_4_ consumption was detected (Levine et al., 2011), taking up to 80 years to recover to pre-land use change levels. It has been demonstrated that the decrease in methane uptake in agricultural soils is due to the destruction of the soil physical structure (e.g., plowing, soil compaction), disrupting the methane gradients in the soil, which are proposed to be crucial for high affinity atmospheric methane oxidation. Next to this other agricultural practices (e.g., fertilization) have been demonstrated to have detrimental effects on atmospheric methane uptake (Bender and Conrad, 1992; Boeckx et al., 1997; Hiltbrunner et al., 2012). However, a recently published study (Ho et al., 2015) demonstrated strongly enhanced methane uptake rates after the addition of different organic amendments (e.g., compost, sewage sludge), to different agricultural soils. The observed rates of uptake were even comparable to the ones from well-aerated forest soils. Shackley et al. observed a similar effect upon addition of biochar which improved the GHG balance by reducing N_2_O and CH_4_ emissions from soil (Shackley et al., 2016). These findings are further supported by another study which showed that the use of organic fertilizers (in this case biochar and compost) influence microbial processes which resulted in alterations of soil nutrient cycles thereby affecting agricultural properties (Ye et al., 2016). Furthermore, the addition of plant-derived C compounds from external sources such as biochar or composts can increase soil C availability and may result in higher net CO_2_ removals from the atmosphere (Paustian et al., 2016) thereby lowering the global warming potential (GWP) (Järveoja et al., 2016). Compared to fresh organic residues, mineralization of compost is slower after addition to soil, leading to a several fold greater mean residence time (Ryals et al., 2015). Ho et al. (2015) postulated that a well-balanced mix of different fertilizers could have a positive effect on GHG balance considering the creation of conditions for methanotrophs to take up atmospheric methane while at the same time keeping carbon dioxide and nitrous oxide emissions to a minimum by providing a greater variety of C- and N-compounds to the microorganisms. However, not all organic fertilizers are suitable to serve this purpose, since in single application only a few organic residues showed the capability to increase soil CH_4_ uptake and keep CO_2_ and N_2_O emissions to a minimum (Ho et al., 2017). However, to develop a strategy to reduce GHG emission from agricultural soils without decreasing crop yield requires understanding of the underlying mechanisms of how organic fertilizers influence GHG. This study aims to answer the following research questions: What is the influence of a combination of organic amendments (compost, digestate, sewage sludge, and cover crop residues) on the GWP of agricultural soils? We hypothesize that methane uptake is stimulated while CO_2_ and N_2_O emissions are kept to a minimum compared to un-amended soil by application of mixes of organic amendment and mineral fertilizers. We test these hypotheses by performing soil incubations with various combinations of organic and mineral fertilizers and following GHG dynamics as well as soil chemistry and microbial functional gene abundance.

## Materials and Methods

### Site Description, Soil Sampling, and Residues

The soil was collected in May 2017 at the research station of Wageningen University in Lelystad, the Netherlands (52°32′26.4′′N, 05°33′34.7′′E) representing a clay soil. The field was planted with onions and left fallow after harvest before sampling. Previously, soil physical-chemical properties have been determined (Ho et al., 2015). The upper 10 cm of the soils was collected in May 2017 from 1 × 1 m using a shovel. The soil was air-dried at room temperature before being sieved (2 mm). The residues included in this study comprised materials with a broad C:N ratio ranging from 4.85 to 22.39 (Table 1) and were selected based on their CH_4_ uptake performance (compost and sewage sludge) (Ho et al., 2015) or their common usage as bio-based additives in agricultural soil. The residues were air-dried at 30°C, the sewage sludge (S), digestate (D), and the cover crop residues (in the following referred to as CC residues) powder mixture were crushed and ground (< 2 mm) (Jaw Crusher Type BB-1/2, Aartselaar, Belgium). Both composts (C1 and C2) were broken down and sieved (< 6 mm), while the CC residues were cut with a scissor to smaller pieces (< 3–5 cm). Both the dried soils and residues were thoroughly mixed and sieved as per treatment prior to setup of the experiment to ensure standardized initial incubation conditions.

**Table 1:** Amendment description, total C and N contents of amendment and soil.

**Soil/residues**	**Total C (μg C mg dw sample^**−1**^)**	**Total N (μg C mg dw sample^**−1**^)**	**C:N**	**Description (source/location)**
Soil	16.44 ± 0.34	1.12 ± 0.07	14.76	Clay soil from an agricultural field with onions as the last crop (Lelystad, The Netherlands)
Sewage Sludge	202.74 ± 12.82	41.81 ± 1.80	4.85	Sampled from an anaerobic digester after sludge thickening (Vallei Veluwe, The Netherlands)
Digestate	290.07 ± 14.14	24.59 ± 1.64	11.82	Residue product of biogas formation from manure (ACRRES, The Netherlands)
Compost1	145.68 ± 39.07	11.08 ± 2.19	13.04	Mature compost derived from organic materials e.g., plant clippings and grass (Attero, The Netherlands)
Compost2	118.40 ± 13.77	6.25 ± 0.65	18.96	Van Iersel fungal dominant humic compost. Basic ingredient is wood shreds (Soiltech, The Netherlands)
CC residue mixture	347.02 ± 15.78	15.50 ± 1.78	22.39	Consist of *Brassica carinata, Trifolium incarnatum, Secale cereal* collected from a field in November 2016 (Joordens, The Netherlands)

### Experimental Setup for *in situ* GHG Flux Measurements

The soil (200 g dry weight) and residues were mixed with a spoon in a pot and put in an incubation bottle (500 mL volume), deionized water was added to 65 or 40% of soil water holding capacity, respectively. The residue addition to the soil corresponded to a rate of either 20-ton ha^−1^, which is typically used in agricultural practice (Diacono and Montemurro, 2010), or 5-ton ha^−1^, which is the maximum amount of cover crop biomass incorporated in agricultural fields in spring. Incubation was performed using three replicates for each treatment in a climate chamber at 15°C (mean annual temperature in the Netherlands is 10°C) in the dark for ~1 month (for 28 days). Water loss, measured by weight, was compensated weekly. Periodically (0, 1, 3, 7, 14, 21, 28 d) methane, nitrous oxide and carbon dioxide fluxes were measured under ambient air by closing the bottles tightly with a lid for 3 h and measuring directly after closing, after 1.5 h and after 3 h. At every time point 20 mL of the headspace was withdrawn and stored in exetainers (5.9 mL) vials (Labco Limited, Lampeter, UK). The first 8 ml of sample was used to flush the exetainer, followed by 12 ml sample introduced into the exetainers creating a 2 bar overpressure. Introduction of the sample (1 ml) into the GC was by an autosampler (TriPlus RSH, Thermo Fisher Scientific, Bleiswijk, The Netherlands) connected to a gas chromatograph (GC1300, Thermo Fisher Scientific) equipped with a Methanizer and a Flame Ionization Detector (FID) to detect CH_4_ and CO_2_, an electron capture detector (ECD) for detection of N_2_O and two sets of a pair Rt-Q-Bond capillary columns (L; 15 m and 30 m, ID; 0.53 mm, Restek, Interscience, Breda, The Netherlands). Helium was used as a carrier gas, and oven temperature was set at 80°C. Five different concentrations of CH_4_ (0.1, 0.2, 0.6, 1.2, 2 ppm), CO_2_ (100, 200, 600, 1,200, 2,000 ppm), and N_2_O (0.05, 0.1, 0.3, 0.6, 1.0 ppm) from a gas mixture (2 ppm CH_4_, 2,000 ppm CO_2_, 1 ppm N_2_O) (Linde AG, Velsen-Noord, The Netherlands) were used as a standard. If higher concentrations of CO_2_ and N_2_O were measured, additional single gas calibration gases (Linde AG) of the respective gases (CO_2_: 4,000 and 10,000 ppm; N_2_O: 10 and 100 ppm) were used. Chromeleon™ Chromatography Data System 7.1 (CDS, Thermo Fisher Scientific) Software was used to analyse the obtained gas chromatograms from the GC and was used to calculate the standard curves. The gas flux rates were determined by linear regression from the three time points. All fluxes with a *R*^2^ < 0.70 were discarded.

### Measuring Methane Oxidation and Organic Matter

To determine near atmospheric soil methane emission or uptake under influence of the different amendments after 7, 14, 21, and 28 d, the bottles were closed for 6 days and ~10 ppm CH_4_ was added to the headspace. CH_4_ decrease was measured every day in duplicates from each bottle using an Ultra GC gas chromatograph (Interscience, Breda, The Netherlands) equipped with a Flame Ionization Detector (FID) and a Rt-Q-Bond (L; 30 m, ID; 0.32 mm, Restek, Interscience) capillary column. Helium was used as a carrier gas, and oven temperature was set at 80°C. Chromeleon™ Chromatography Data System 7.1 (CDS, Thermo Fisher Scientific) Software was used to analyse the obtained gas chromatograms from the GC.

### Sample Storage and Soil Organic Matter Measurements

After finishing the incubation ~10 g of soil samples were stored at −20°C for later DNA extractions. Another ~50 g of soil was dried at 30°C and stored for soil nutrient determination. To measure the soil organic matter content after incubation, 10–15 g of soil was dried in a porcelain cup at 105°C for 1 day. Afterwards, the dried sample was burned in an oven at 430°C for another day, both times the sample was weighed. To calculate the organic matter content per g 100 g^−1^ dry soil the following formula was used: 100^*^ (g dry soil—g ashed soil)/g dry soil.

### DNA Extraction and qPCR Assays

DNA was extracted using the DNeasy PowerSoil Kit (Qiagen, Venlo, The Netherlands) according to manufacturer's instruction. We performed qPCR assays targeting *amoA* for ammonium oxidizing Achaea (AOA) and bacteria (AOB), *nifH* (N-fixers), *nosZ* clade I/II (denitrifiers), *mcrA* (methanogens), *pmoA* (methane oxidizers), 16S rRNA gene for Archaea and Bacteria as well as the 18S rRNA gene of fungi. Each assay was performed in duplicate for each DNA extract with primers, primer concentration, and PCR profiles as shown in Supplementary Table 1. Briefly, each qPCR (total volume 20 μl) for all assays consisted of 10 μl 2 × SensiFAST SYBR (BIOLINE, Alphen aan den Rijn, The Netherlands), 1 μl of forward and reverse primers each (10 pmol μl^−1^; Sigma-Alderich, Zwijndrecht, The Netherlands), 1 μl bovine serum albumin (5 μg μl^−1^; Invitrogen, Breda, The Netherlands), 4.5 μl DNase- and RNase-free water and 2.5 μl diluted template DNA. The qPCR for the EUBAC(bacterial 16S rRNA gene) assay (total volume 15 μl) consisted of 7.5 μl 2 × SensiFAST SYBR (BIOLINE), 0.75 μl of forward and reverse primers each (5 pmol μl^−1^; Sigma-Alderich), 1.5 μl bovine serum albumin (5 μg μl^−1^; Invitrogen), 1.5 μl DNase- and RNase-free water, and 3 μl diluted template DNA. Standard curves were obtained using serial 10-fold dilutions of a known amount of plasmid DNA from different pure cultures representing the target gene fragment (10^8^–10^1^ gene copies) containing the respective gene fragment. The qPCR was performed with an iCycler IQ5 (Applied Biosystem, Carlsbad, CA, USA). Negative controls were always run with water instead of template DNA. PCR reactions were done with 1:20 and 1:60 diluted DNA extracts. Amplification efficiencies for all assays were between 79 and 98% with *R*^2^ values between 0.969 and 0.995. Amplicon specificity was inferred from the melt curve.

### Statistical Analyses of Collected Data

All statistical analyses were done using R version 3.0.1 (R Development Core Team, 2013). The mean total GHG fluxes, the GWP, the organic matter loss and abundance of the different functional marker genes were tested for normality by Kolmogorov–Smirnov test and for homogeneity of variance by Levene's test. If necessary, normal distribution was achieved by log-transformation of the data. Treatment effects and differences between means were assessed using one-way ANOVA followed by Tukey's *post-hoc* test. All levels of significance were defined at *P* < 0.05.

## Results

### GHG Flux Measurements

The fluxes of the three major GHG (CH_4_, CO_2_, N_2_O) from the soils amended with the organic amendments were measured continuously through the experiment at different level of SM and different applied concentrations of organic amendments. An overview about values of the different GHG as well as the calculated GWP_100yr_ for the different samples is shown in Table 2.

**Table 2:** Overview of mean total CH_4_. CO_2_, N_2_O, and calculated GWP_100yr_ values of the different organic amendments, amounts, and soil moisture concentration that were used.

**OA**	**Amount [t/ha]**	**Mean total CH**_**4**_**-C [μg kg soil**^**−1**^**]**		**Mean total CO**_**2**_**-C [mg kg soil**^**−1**^**]**		**Mean total N**_**2**_**O-N [mg kg soil**^**−1**^**]**		**GWP**_**100yr**_ **[mg CO**_**2**_**-C kg soil**^**−1**^**]**	
		**40%SM**	**65%SM**	**40%SM**	**65%SM**	**40%SM**	**65%SM**	**40%SM**	**65%SM**
Un-amended	None	75 ± 50.2	83 ± 18.0	958 ± 71.3	1,068 ± 132.6	0.015 ± 0.008	0.063 ± 0.034	959 ± 71.2	1,072 ± 134.8
C1	20	100 ± 28.6	70 ± 53.7	1830 ± 117.5	2,357 ± 256.4	0.057 ± 0.008	0.290 ± 0.052	1834.4 ± 117.1	2375.9 ± 257.7
	5	142 ± 123.4	125 ± 23.8	818 ± 464.6	1,717 ± 73.3	0.002 ± 0.015	0.0414 ± 0.014	819 ± 464.8	1,744 ± 79.9
C2	20	91 ± 27.1	88 ± 42.7	1,058 ± 27.3	1,586 ± 14.3	0.013 ± 0.001	0.042 ± 0.014	1,060 ± 27.1	1,589 ± 13.7
	5	91 ± 24.1	88 ± 32.5	777 ± 35.2	1,426 ± 75.7	0.009 ± 0.005	0.425 ± 0.457	778 ± 34.9	1,453 ± 13.6
Cut CC	*20*	134 ± 52.2	70 ± 37.4	32,372 ± 2762.6	46,157 ± 1289.2	13.651 ± 1.879	16.877 ± 2.182	33,218 ± 2670.6	47,201 ± 1381.7
	*5*	70 ± 46.5	84 ± 22.8	6,303 ± 1057.9	11,689 ± 1220.0	0.107 ± 0.033	5.482 ± 2.244	6,310 ± 1059.5	12,028 ± 1358.8
Powder CC	20	118 ± 27.9	70 ± 30.5	20,098 ± 1538.7	26,177 ± 1006.6	19.345 ± 3.967	6.397 ± 2.67	21,295 ± 1422.1	26,688 ± 1031.1
	5	82 ± 19.1	109 ± 47.2	5,286 ± 1205.6	7,996 ± 1429.6	0.031 ± 0.013	4.666 ± 5.425	5,289 ± 1205.9	8,236 ± 1143.8
Digestate	20	80 ± 40.3	−77 ± 21.3	4,554 ± 780.5	6,583 ± 316.5	0.280 ± 0.335	6.204 ± 2.207	4,572 ± 800.9	6,966 ± 183.7
	5	104 ± 53.8	107 ± 94.0	2,322 ± 277.4	2,750 ± 490.8	0.032 ± 0.049	2.029 ± 0.457	2,325 ± 279.4	2,877 ± 129.9
D+C1	20	48 ± 32.1	30 ± 25.7	2,734 ± 177.8	3,807 ± 348.0	0.105 ± 0.060	2.259 ± 0.189	2,741 ± 180.8	3,947 ± 359.0
	5	46 ± 28.8	18 ± 55.1	1,560 ± 383.7	2,070 ± 95.6	−0.033 ± 0.093	1.059 ± 0.30	1,558 ± 389.5	2,135 ± 84.4
D+C2	20	75 ± 74.9	−70 ± 61.1	2,135 ± 34.1	3,848 ± 1239.4	0.056 ± 0.049	8.183 ± 10.67	2,139 ± 31.9	4,354 ± 1895.3
	5	57 ± 15.4	12 ± 29.6	1,118 ± 208.5	1,711 ± 103.1	0.028 ± 0.011	0.586 ± 1.211	1,120 ± 208.1	1,747 ± 70.0
S+C1	20	53 ± 23.9	−40 ± 44.5	4,884 ± 362.0	6,057 ± 2144.8	1.485 ± 0.271	28.589 ± 15.345	4,976 ± 345.2	7,825 ± 3042.5
	5	85 ± 40.8	9 ± 38.9	1,853 ± 137.3	2,527 ± 63.0	0.263 ± 0.190	9.306 ± 4.354	1,870 ± 130.0	3,102 ± 289.9
S+C2	20	107 ± 71.2	16 ± 23.8	4,561 ± 336.3	6,170 ± 209.3	0.707 ± 0.047	32.501 ± 3.094	4,605 ± 336.1	8,178 ± 399.2
	5	88 ± 24.1	62 ± 64.5	1,648 ± 203.7	2,266 ± 186.1	−0.016 ± 0.091	8.756 ± 2.053	1,647 ± 208.7	2,808 ± 313.5

*GWP_100yr_ calculations derived from the cumulative CH_4_ (Supplementary Figure 1), CO_2_ (Supplementary Figure 2), and N_2_O (Supplementary Figure 3) fluxes. OA, organic amendments; un-amended, soil without organic amendment; C1, compost1; C2, compost2; cut CC, cut cover crop residue mixture; powder CC, powder cover crop residue mixture mix; D+C1, digestate + compost1; D+C2, digestate + compost2; S+C1, sewage sludge + compost1; S+C2, sewage sludge + compost2*.

#### CH_4_

**Figure 1:**
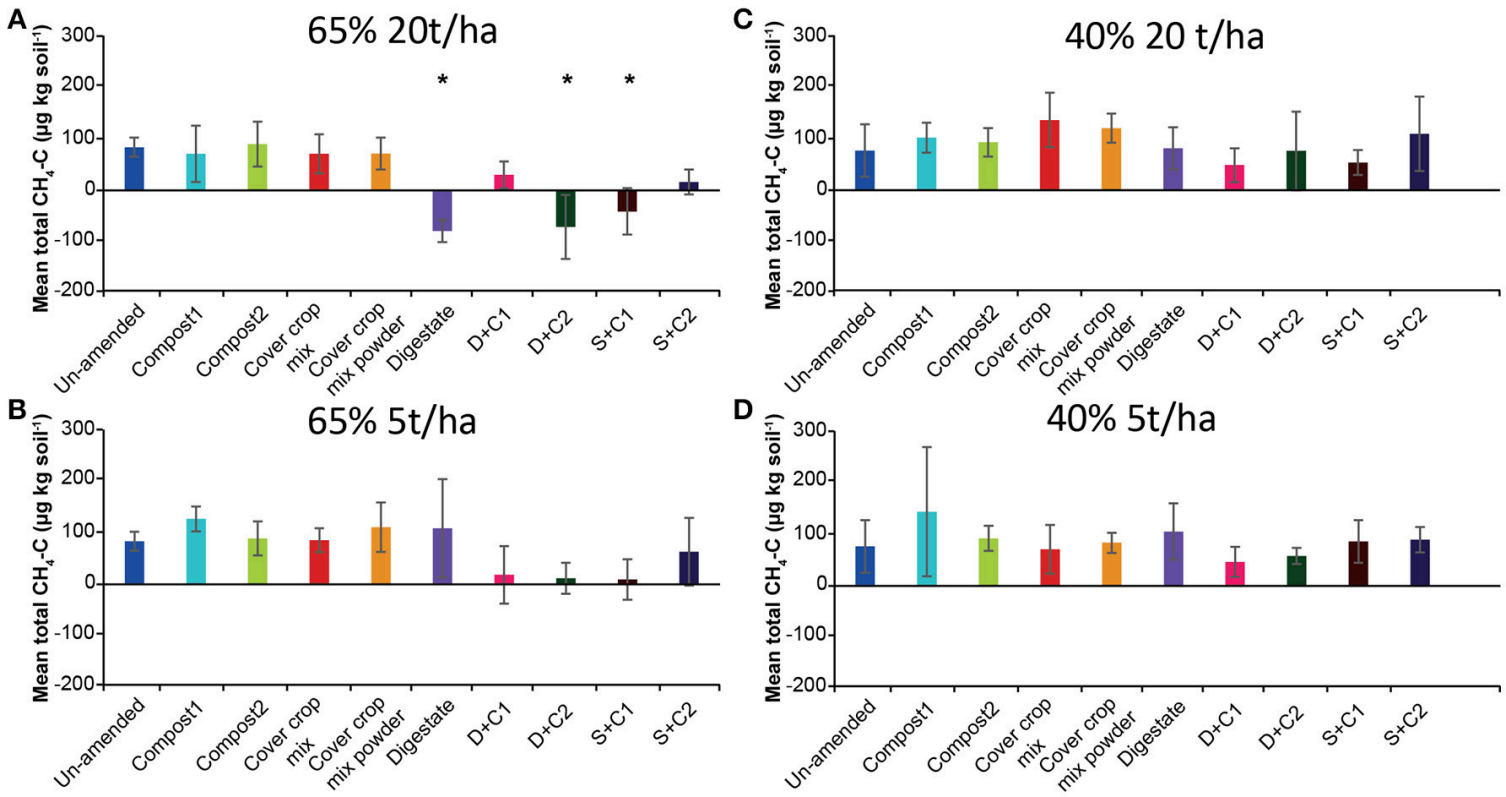
Mean total CH_4_ emitted or consumed over the period of 28 d in un-amended clay soil and after amendments with compost1, compost2, CC residues mixtures, digestate, digestate+compost1, digestate+compost2, sewage sludge+compost1, and sewage sludge+compost2 (mean ± SD; *n* = 3) at **(A)** high amount (20 t/ha) and high water content (65%), **(B)** low amount (5 t/ha) and high water content, **(C)** high amount and low water content (40%), and **(D)** low amount and low water content, derived from the cumulative CH_4_ (Supplementary Figure 1) fluxes. Asterisk (*) indicate significant differences in the mean total CH_4_ fluxes between the soils with organic amendments and the un-amended soil within the four separate superordinate treatments (ANOVA: *P* < 0.05).

The CH_4_ flux measurements under 65% SM (Supplementary Figures 1A,B) showed variation over time considering uptake or emission of CH_4_. Both amounts of organic amendments applied (5 and 20 t/ha) led to similar fluxes during the incubation without fluctuation. However, total CH_4_ fluxes (Figures 1A,B) varied between treatments, mostly releasing CH_4_ over time irrespective of the amount of organic amendment used. Only three amendments (digestate, D + C2, S + C1 at 20 t/ha) led to increased methane uptake. Under 40% SM, minor fluctuations in CH_4_ fluxes over time were detected with both organic amendment amounts (Supplementary Figures 1C,D). Calculated mean cumulative CH_4_ fluxes (Figures 1C,D) demonstrated that all samples emitted CH_4_ during the incubation.

#### CO_2_

**Figure 2:**
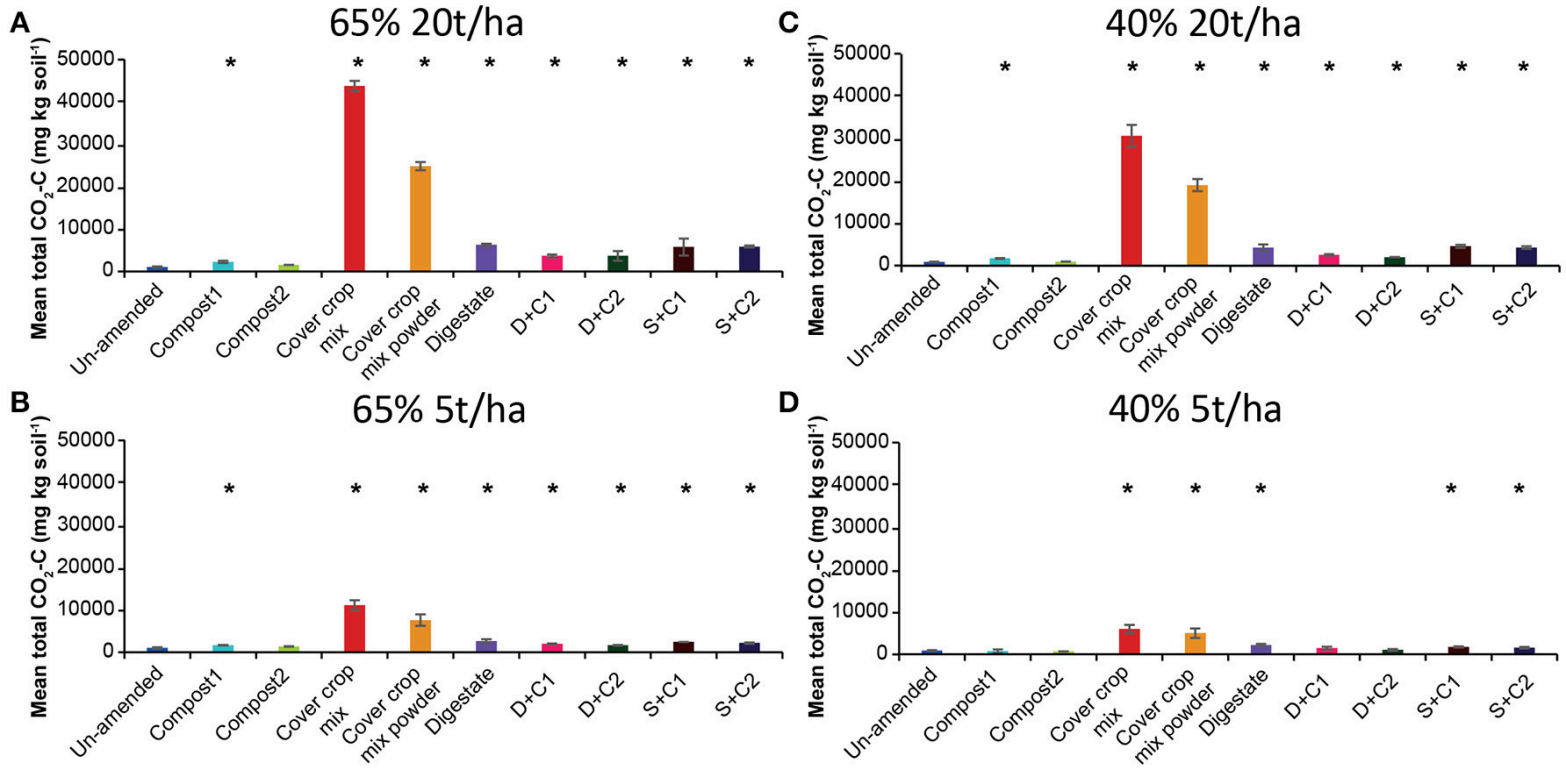
Mean total CO_2_ emitted over the period of 28 d in un-amended clay soil and after amendments with compost1, compost2, CC residues mixtures, digestate, digestate+compost1, digestate+compost2, sewage sludge+compost1, and sewage sludge+compost2 (mean ± SD; *n* = 3) at **(A)** high amount (20 t/ha) and high water content (65%), **(B)** low amount (5 t/ha) and high water content, **(C)** high amount and low water content (40%), and **(D)** low amount and low water content, derived from the cumulative CO_2_ (Supplementary Figure 2) fluxes. Asterisk (*) indicate significant differences in the mean total CO_2_ fluxes between the soils with organic amendments and the un-amended soil within the four separate superordinate treatments (ANOVA: *P* < 0.05).

Measured CO_2_ fluxes under 65% SM (Supplementary Figures 2A,B) showed the same trends, irrespective of the amounts of organic amendment applied. Highest CO_2_ fluxes were observed for cut and powdered cover crop residues, respectively, followed by digestate and the sewage sludge + compost 2 combination. Independent of the amount applied, cut as well as powdered CC residues continuously released CO_2_ over the complete incubation. Both types of compost led to the lowest CO_2_ fluxes among the organic amendments used and were comparable or lower than the CO_2_ fluxes of the un-amended soil. The mean cumulative CO_2_ fluxes (Figures 2A,B) reflect the dynamics of the CO_2_ fluxes over time and treatments (Supplementary Figures 2A,B). Highest CO_2_ emissions were observed for cut CC residue material, followed by powdered CC residue, digestate, and the sewage sludge amendments. This was true for both tested amounts. Highest CO_2_ fluxes under 40% SM were always observed for cut CC residue material followed by powdered CC residues, digestate and the two sewage sludge treatments (Supplementary Figures 2C,D). While high amounts of CC residues showed emission of CO_2_ over the whole incubation period, no emissions were detected after 21 d with low amounts. Similarly, cumulative CO_2_ fluxes (Figures 2C,D) were always lower with lower amounts of organic amendments, the extent of which differed between the type of organic amendment. While both cover crop residue treatments were 4- to 5-fold higher, all other organic amendments were only 1.4- to 2.7-fold higher when 20t/ha was applied.

Lower SM always lead to lower CO_2_ fluxes when same amounts organic amendments were applied.

#### N_2_O

**Figure 3:**
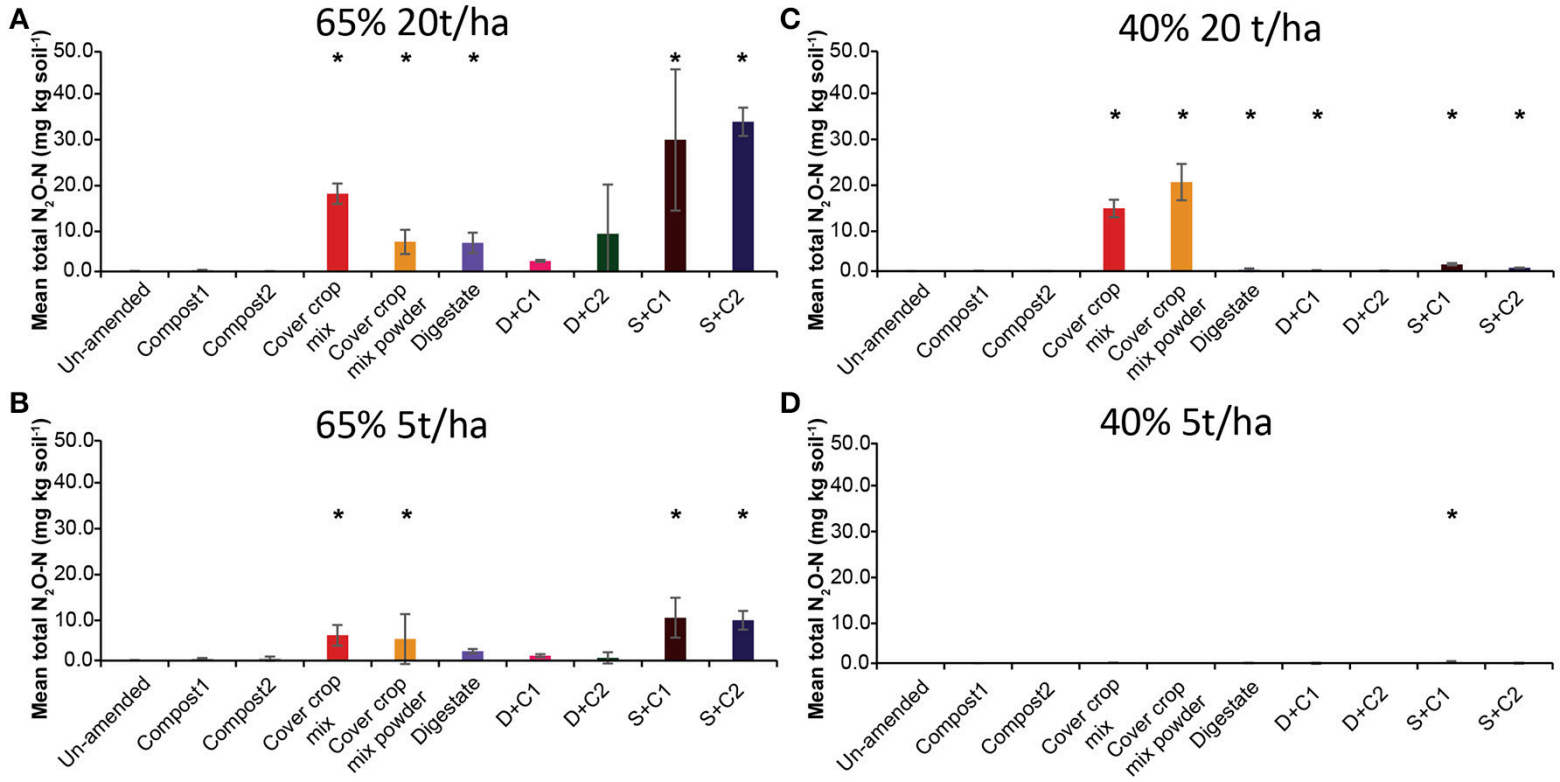
Mean total N_2_O emitted over the period of 28 d in un-amended clay soil and after amendments with compost1, compost2, CC residues mixtures, digestate, digestate+compost1, digestate+compost2, sewage sludge+compost1, and sewage sludge+compost2 (mean ± SD; *n* = 3) at **(A)** high amount (20 t/ha) and high water content (65%), **(B)** low amount (5 t/ha) and high water content, **(C)** high amount and low water content (40%), and **(D)** low amount and low water content, derived from the cumulative N_2_O (Supplementary Figure 3) fluxes. Asterisk (*) indicate significant differences in the mean total N_2_O fluxes between the soils with organic amendments and the un-amended soil within the four separate superordinate treatments (ANOVA: *P* < 0.05).

Both sewage sludge combinations showed the highest N_2_O flux rates at 65% SM, regardless of the applied amounts of organic amendments, followed by digestate and cut CC residue material (Supplementary Figures 3A,B). Both composts, as well as the un-amended soil, showed almost no N_2_O fluxes. In general, 20 t/ha led to higher overall measurable N_2_O fluxes. These findings are also underlined by the cumulative N_2_O fluxes (Figure 3). The N_2_O fluxes of both sewage sludge combination, digestate, digestate + compost 1, and both CC residue mixtures were 2- to 4-fold lower with 5 t/ha. The digestate + compost 2 amendment showed a 13-fold reduction, while the un-amended and both single compost applications did not lead to any N_2_O emission at all. After 14 d of incubation both combinations of digestate with compost at an application rate of 5 t/ha resulted in lower N_2_O emissions.

Only low N_2_O emissions were detected at 40% SM (Supplementary Figures 3C,D). All organic amendments applied at a rate of 5 t/ha showed no N_2_O emissions during the complete incubation period while at 20 t/ha only small amounts of N_2_O were released in the first 14 d of incubation. After 14 d both CC residue amendments (cut and powdered) showed a rapid increase in N_2_O emissions, which peaked at day 21. After 28 d the cut CC residues still released N_2_O from the soil, while the powdered CC residue enabled soil N_2_O uptake from this point onward.

#### GWP Analyses

We derived the GWP in mg CO_2_ equivalent per kg soil by combining the cumulative CH_4_, CO_2_, and N_2_O flux (Supplementary Figures 1–3). In these calculations, the GWP value for CH_4_ and N_2_O are considered to be 28 and 265, respectively over a hundred- year time frame, while the GWP value for CO_2_ is considered to be 1 (IPCC, 2014).

**Figure 4:**
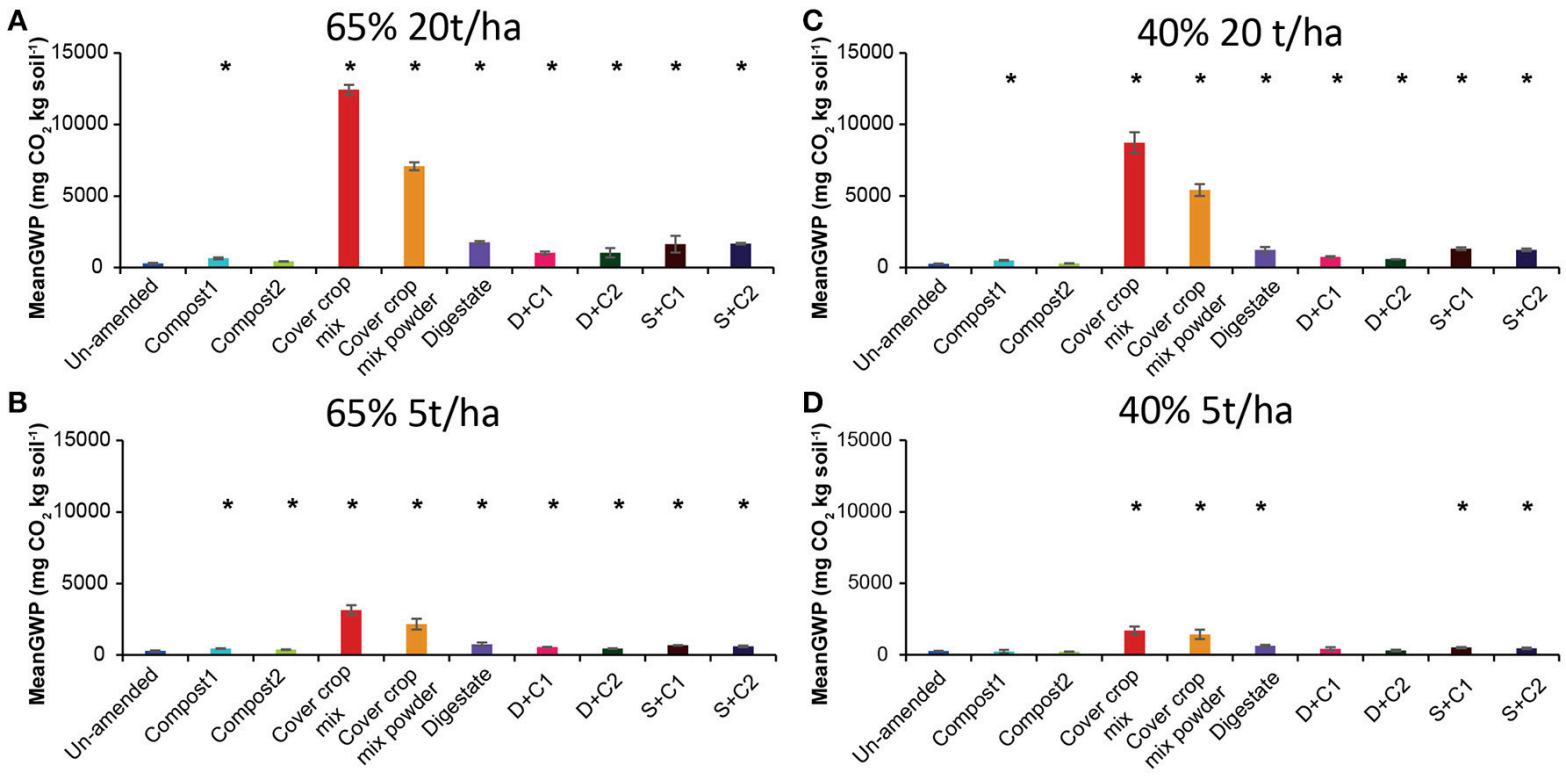
Mean global warming potential (GWP) over the period of 28 d in un-amended clay soil and after amendments with compost1, compost2, CC residues mixtures, digestate, digestate+compost1, digestate+compost2, sewage sludge+compost1, and sewage sludge+compost2 (mean ± SD; *n* = 3) at **(A)** high amount (20 t/ha) and high water content (65%), **(B)** low amount (5 t/ha) and high water content, **(C)** high amount and low water content (40%), and **(D)** low amount and low water content, derived from the cumulative CH_4_ (Supplementary Figure 1), CO_2_ (Supplementary Figure 2), and N_2_O (Supplementary Figure 3) fluxes. Asterisk (*) indicate significant differences in the GWP between the soils with organic amendments and the un-amended soil within the four separate superordinate treatments (ANOVA: *P* < 0.05).

The GWP values showed similar trends as the cumulative CO_2_ fluxes, irrespective of the SM and amount of organic amendment (Figure 4). Notably, compost1 and 2 treatments led to lower GWP as compared to un-amended soil with low amounts applied under 40% SM (Figure 4).

#### CH_4_ Fluxes After Addition of 10 ppm CH_4_

CH_4_ fluxes after the addition of 10 ppm CH_4_ at multiple times, did not differ significantly between the four major treatments irrespective of SM and organic amendment rate applied (Supplementary Figure 4). The fluxes in most cases vary between 0 and −0.003 μmol m^−2^ min^−1^, which can be referred to as CH_4_ uptake. At the last sampling point the amendment with compost2 at 40% SM and 5 t/ha increased to an uptake of −0.008 μmol m^−2^ min^−1^, which was the highest uptake measured. However, most organic amendments improve their CH_4_ uptake over time.

### Organic Matter

**Figure 5:**
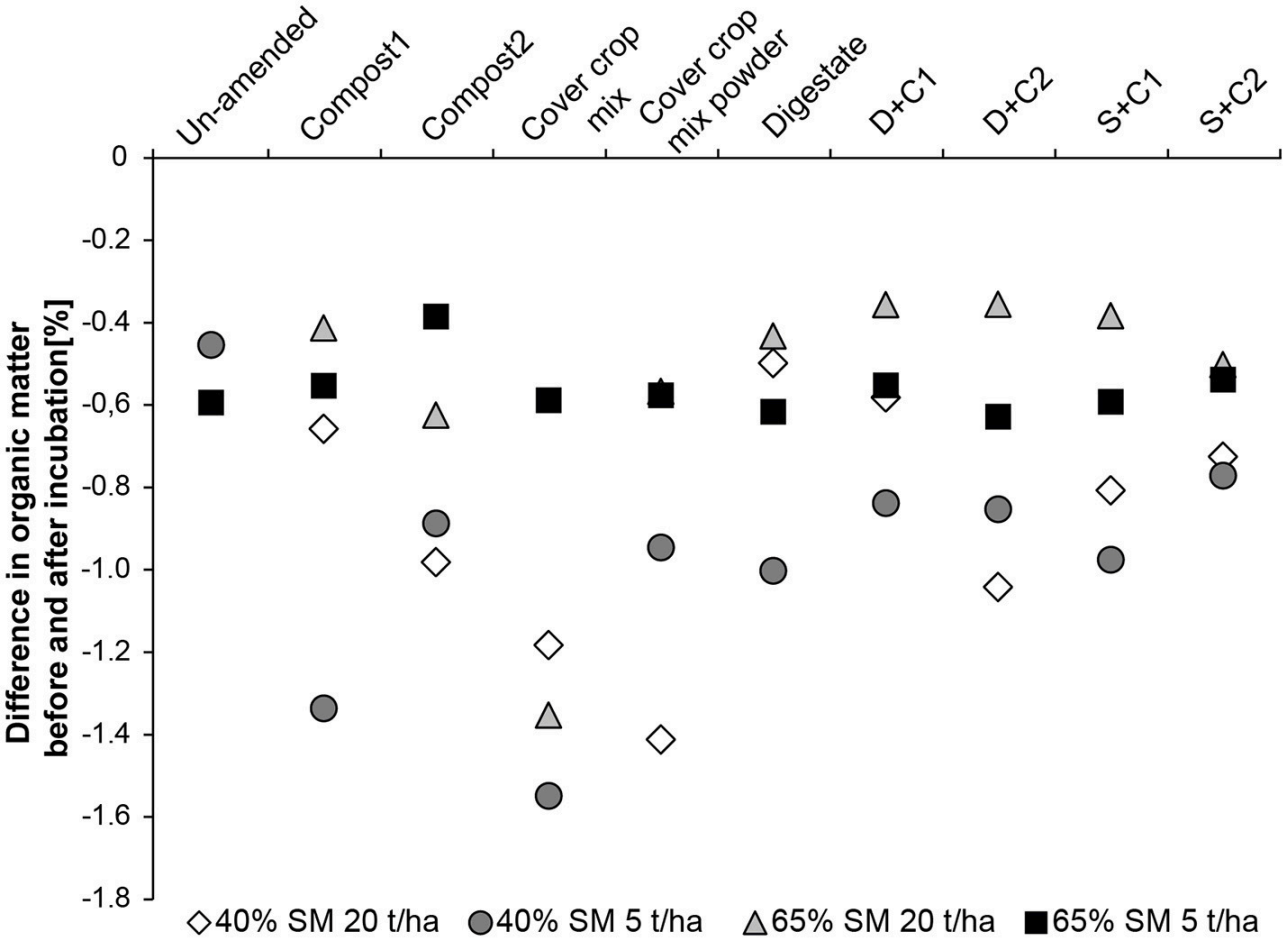
Loss in organic matter content during the incubation period of 28 d in un-amended clay soil and during amendments with compost1, compost2, CC residues mixtures, digestate, digestate+compost1, digestate+compost2, sewage sludge+compost1, and sewage sludge+compost2 (mean ± SD; *n* = 3) at (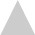) high amount (20 t/ha) and high water content (65%), (■) low amount (5 t/ha) and high water content, (♢) high amount and low water content (40%), and (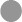) low amount and low water content.

When low amounts of organic amendment are applied at 65% SM, the organic matter loss is constant through all treatments ranging from −0.4 to −0.6% loss of the original OM content which was around 2.5–3% (Figure 5). At high concentration of organic amendments the loss of OM is lower being around −0.4% with exception of the cut CC residue amendment, resulting in 1.4% loss in organic matter. In general incubations at 40% SM lost more organic matter than their counterpart at 65% SM (Figure 5). The lowest losses were observed for digestate, compost1, and D+C1 with a loss of ~-0.55%. These organic amendments are followed by compost2, D+C2, S+C1, and S+C2 with a loss of −0.8 to −1.0% organic matter content. The highest loss could be observed for cut and powdered CC residue mixture with −1.2 and −1.4%, respectively.

### Abundance Analyses of Microbial Groups

To assess changes in the abundance of the microbial communities, the ratio was calculated between gene copy numbers of the analyzed genes in the initial soil and at the end of the incubation. The individual gene copy numbers of all samples analyzed can be found in Supplementary Tables 2, 3.

**Figure 6:**
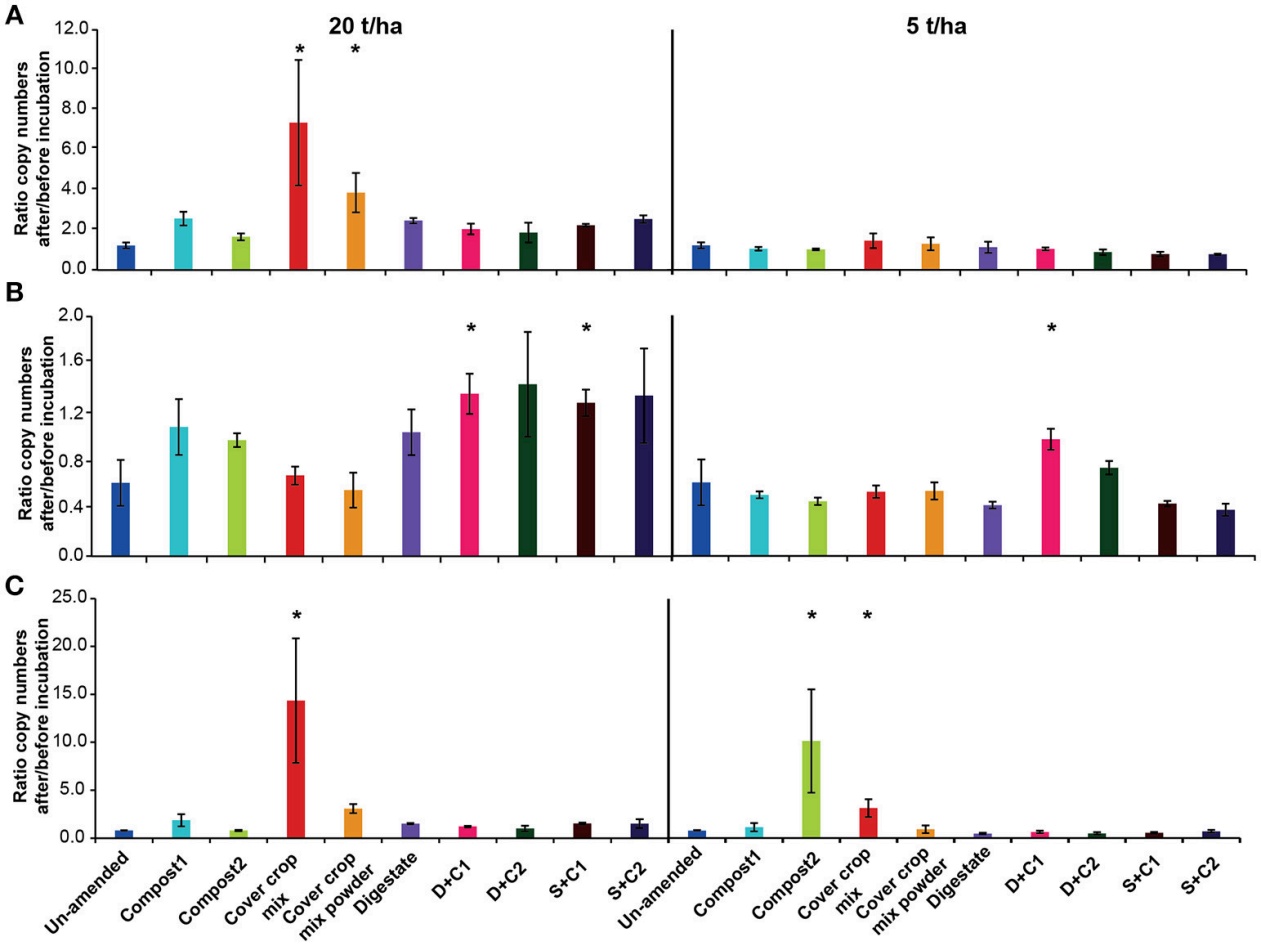
Ratio of the copy numbers of **(A)** bacterial 16S rRNA gene, **(B)** archaeal 16S rRNA gene, and **(C)** fungal 18S rRNA gene after and before an incubation of un-amended clay soil and during amendments with compost1, compost2, CC residues mixtures, digestate, digestate+compost1, digestate+compost2, sewage sludge+compost1 and sewage sludge+compost2 (mean ± SD; *n* = 3) for 28 d. Asterisk (*) indicate significant differences in the ratio of the individual genes in the soils with organic amendments and the un-amended soil within the four separate superordinate treatments (ANOVA: *P* < 0.05).

The overall bacterial abundance stayed either stable or increased over time (Figure 6A), with high amounts of CC residues leading to the highest stimulation in abundance (4- to 7-fold). All other organic amendments at high application rate led to at least to a doubling of bacterial numbers, while numbers in the un-amended remained constant. When applying low amounts of organic amendments, microbial abundances did not change in any of the treatments.

In contrast to the bacterial abundance, archaea communities either remained stable or decreased over the time (Figure 6B). Typically, all digestate combinations, both composts and sewage sludge combinations at high application rate did not lead to change in archaeal abundance, while it decreased in all other treatments.

Overall, fungal abundance was rather constant during the incubation (Figure 6C). However, the cut CC residue mixture led to a 15- and 5-fold increase in fungal abundance at high and low organic amendment application rate, respectively while the 20 t/ha powdered CC residue treatment increased around 3-fold. Compost 2 at low application led a 10-fold in increase. All other treatments at high application rate did not lead to change in fungal abundance.

**Figure 7:**
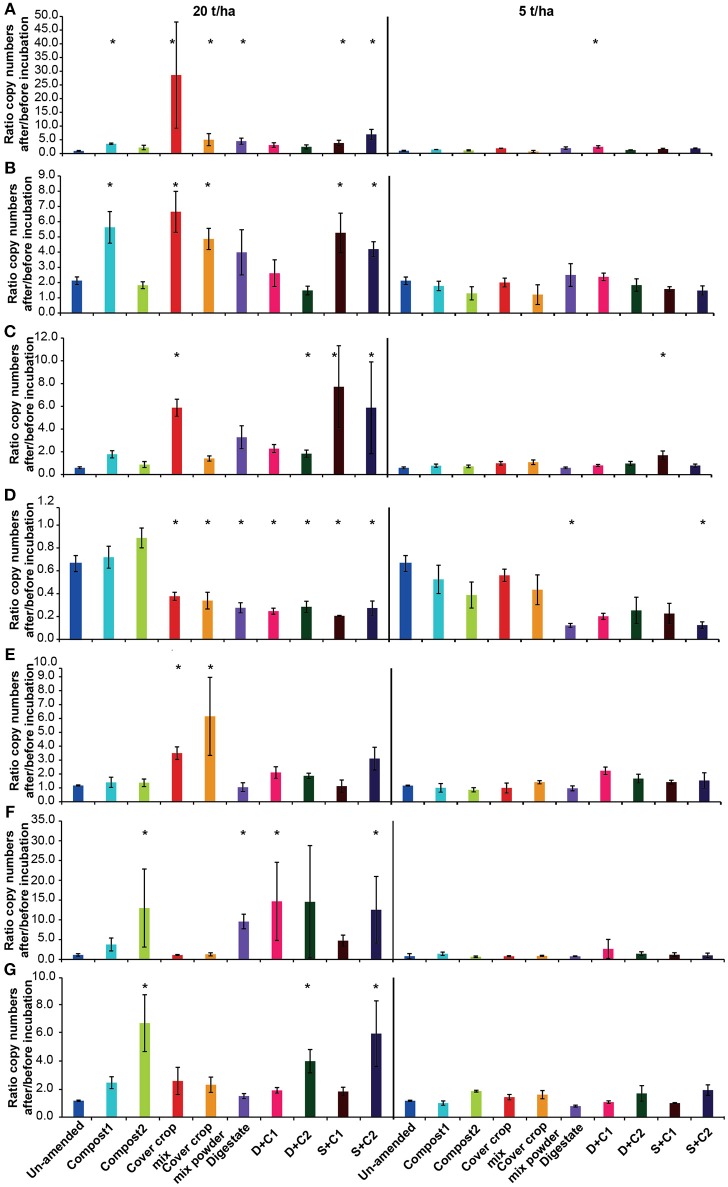
Ratio of the copy numbers of the functional marker genes **(A)** *nosZ* clade I, **(B)** *nosZ* clade II, **(C)** bacterial *amoA*, **(D)** archaeal *amoA*, **(E)** *nifH*, **(F)** *mcrA*, and **(G)** *pmoA* after and before an incubation of un-amended clay soil and during amendments with compost1, compost2, CC residues mixtures, digestate, digestate+compost1, digestate+compost2, sewage sludge+compost1, and sewage sludge+compost2 (mean ± SD; *n* = 3) for 28 d. Asterisk (*) indicate significant differences in the ratio of the individual genes in the soils with organic amendments and the un-amended soil within the four separate superordinate treatments (ANOVA: *P* < 0.05).

For most of the functional marker genes there was no change in the un-amended soil, except for a decrease of AOAs and a doubling of *nosZ* clade II (Figure 7).

Both *nosZ* clades showed an increase in abundance, in all organic amendment-treatments, irrespective of the application rate (Figures 7A,B). While the two clades with low amendments increased mainly between 1.2- and 2.5-fold, a 2- to 7-fold increase was observed with 20 t/ha. The highest increase occurred in the incubation with cut CC residue material with 28-fold in *nosZ* clade I. In general, the *nosZ* clade II was 10- to 100-fold more abundant than *nosZ* clade I (Supplementary Table 2).

At low application rates organic amendments had no effect on the bacterial *amoA* abundance (Figure 7C). At high concentrations, the cut CC residue, both sewage sludge combinations and all treatments with digestate lead to an increase in bacterial *amoA* of 2- to 8-fold (Figure 7C).

In contrast to the abundance of the bacterial *amoA*, archaeal *amoA* abundance decreased in all organic amendment-treatments (Figure 7D). The strongest decrease was observed for the digestate and sewage sludge combinations with both composts, which decreased 3- to 4-fold in both applied concentrations. In all compost, CC residue and digestate amendments AOA gene copy numbers were 2- to 10-fold higher than for AOBs. This is contrast with the sewage sludge treatments, which at low amendment led to higher numbers of AOA, whereas AOBs showed a 2- to 4-fold higher abundance at high organic amendment (Supplementary Table 2).

The abundance of N-fixers in the cut and powdered CC residue mixture increased in the application with 20 t/ha by 3- and 6-fold, respectively (Figure 7E). The only other treatment with a positive effect on the abundance of *nifH* was the sewage sludge + compost 2 amendment, which showed an increase of ~3-fold.

The methanogenic abundance did not changed for both cover crop treatments, but increased 3-fold for compost1, 5-fold for sewage sludge+compost1 and between 10- and 14-fold for the remaining organic amendments at high rates of application while at low rates *mcrA* gene abundance stayed stable (Figure 7F).

Gene copy number of methanotrophs (*pmoA*) increased for all samples with 20 t/ha, except in the digestate amendment, in which no differences to un-amended soil were reported (Figure 7G). The compost2 amendment and the combination with compost2 showed the strongest effect on the copy numbers with a 4- to 6-fold increase. Low organic amendment application rates only showed minor positive effects on the abundance of methanotrophs.

The abundance of the two CC residue amendments at low SM and high organic amendment application behaved very similar for all analyzed genes (Supplementary Table 4). The abundance of the archaeal 16S rRNA gene and archaeal *amoA* dropped by 2-fold, while it stayed stable for *nifH, mcrA*, and *pmoA*. A 5-fold increase was observed for the fungal 18S rRNA gene and *nosZ* clade I for the cut CC residues, while the powder led to a 3- and 2-fold increase, respectively. *nosZ* clade II numbers increase for both CC residue materials around 3-fold. While the cut CC residue material resulted in a 2-fold increase for the bacterial 16S rRNA gene and the bacterial *amoA*, the powdered CC residue material did not show a change for these two genes.

## Discussion

In this study, we investigated the influence of combinations of organic amendments on the GHG balance and the CH_4_ uptake as well as on dynamics of different soil microbial groups that are involved in producing or reducing GHGs in agricultural soil. Several combinations of amendments led to reductions of CO_2_, CH_4_, and/or N_2_O emissions compared to un-amended soil. Most optimal GHG balance was obtained by compost amendments, which resulted in similar overall GHG balance as compared to the un-amended soil. Additionally, we could observe a strong increase in microbial communities involved in GHG consumption in all amendments, with the strongest increase associated with cover crop residue mixtures.

### GHG Dynamics and GWP in Relation to Different Organic Amendments and Manipulation of Soil Moisture

#### CH_4_

We did not observe significant uptake of CH_4_ in any of our samples except for digestate (D), D+C2, S+C1 at high SM and high application rate, which led to CH_4_ uptake over the complete incubation period (Figure 1). However, the *in vitro* methane uptake capacity at near atmospheric (i.e., 10 ppm) methane concentrations increased in all samples over time. As proposed by Ho et al. (2015), it seems that the methanotrophic community needs elevated methane to gear up the enzyme machinery. A similar result was found in rice soils where high methane concentration spikes were necessary to induce atmospheric methane uptake (Cai et al., 2016). Especially the 5 t/ha compost2 treatment under 40% SM showed a very strong improvement in CH_4_ uptake at the end of the incubation. Potentially, the release of rare earth metals (e.g., La, Ce, Nd), which are stored in the compost (La ~2.2 μg g^−1^; Ce ~3.8 μg g^−1^; Nd ~2.2 μg g^−1^; El-Ramady, 2011) stimulated the CH_4_ uptake (Keltjens et al., 2014). Recent studies found that for some methanotrophs rare earth metals are essential as cofactors in the active center of an alternative methanol dehydrogenase (Keltjens et al., 2014; Pol et al., 2014; Shiller et al., 2017). Furthermore, it was shown that the La-dependent methanol dehydrogenase can also be more efficient hydrolytic catalysts because they are stronger Lewis acids (electrophilic electron acceptor) than the Ca dependent one (Lim and Franklin, 2004). This La-dependent methanol dehydrogenase which can also be found in the newly isolated atmospheric CH_4_ oxidizer belonging to the USCα cluster (Pratscher et al., 2018). However, all studies with rare earth metals and their effect on CH_4_ oxidation were performed in liquid cultures. Another possible explanation for the increase in CH_4_ oxidation rates at the end of the incubation in compost2 incubations, could be its relatively low C- and N-content in comparison to the other organic amendments. This could lead to higher amounts of essential substrates (O_2_) or lower amounts of inhibiting compounds (e.g., NH${}_{4}^{+}$) for methane oxidation (Conrad and Rothfuss, 1991; Bender and Conrad, 1992; Malyan et al., 2016). In contrast, the higher amount of C- and N-compounds in the other organic amendments could result in a reduced or delayed start of CH_4_ oxidation. Furthermore, it is known that compost could lead to an increase in the soil's cation binding capacity (Epstein et al., 1976), leading to lowering of the availability of ammonium ions, potentially inhibiting the particulate methane monooxygenase (Singh and Seneviratne, 2017).

#### CO_2_

The first addition of water induced a direct emission of CO_2_ from the soil in samples with organic amendments. The extend of these CO_2_ emissions is strongly dependent on the amendment used. The lowest CO_2_ emissions were obtained with both compost amendments, showing similar values as the un-amended soil, where the fungal based compost emitted less CO_2_ than the compost from green cut materials (Table 2). The reason for this could be a low total C concentration together with not easily degradable C-compounds (Ryals et al., 2015). Based on this it seems that the different material of the compost can contribute better or worse to reducing GHG, which would need further analyses.

The decrease respiration in organic matter added through the experiment did not correlate with most of the CO_2_ fluxes. Only the CO_2_ fluxes under moist conditions (*R*^2^ = 0.633) and high amount of organic amendment (*R*^2^ = 0.783) correlated with the decrease of organic matter. This is in accordance with our previous study (Ho et al., 2017), demonstrating that C:N alone is not a good predictor of amendment effects on GHG fluxes. In this study the organic amendment with the highest C:N ratio was the fungal based compost which showed the lowest measurable CO_2_ fluxes of all organic amendments. However, the highest measured CO_2_ fluxes were emitted by both CC residue mixtures which indeed have the second highest C:N ratio. We observed a correlation between the total C concentration measured in the organic amendments and the CO_2_ fluxes. The quality and composition of the amendments, seem to be more important for influencing the CO_2_ fluxes. For example the sewage sludge+compost2 amendment has the same total C-content as compost1, but emitted 4-fold higher CO_2_ fluxes. In accordance with this, digestate has a lower total C concentration compared to CC residue material, but emitted 15-fold less CO_2_. One explanation is that the digestate is not as easily degradable as the plant material for the microorganisms, since its origin is already anaerobically digestaed manure. It was already shown that CO_2_ respiration from digestate is highly dependent on the initial source from which the digestate is produced, which led to a broad range of CO_2_ respiration rates (Alburquerque et al., 2012). According to our results, this statement can be extended to a variety of organic amendments.

Surprisingly, we saw a second peak of increased CO_2_ emission after 21 d in almost all treatments. This may be explained by the fact after 14 d substrates which are more difficult to degrade are reduced to a more accessible form of shorter chain molecules. Succession in microbial community composition may be involved which can take place in just a short period of time (13–15 d) following amendment with organic residues as shown by Ho et al. (2017). Additionally, changes in soil parameters (e.g., O_2_ availability, N availability) may cause a second phase of CO_2_ respiration due to alleviation of initial limitations.

#### N_2_O

Surprisingly, the highest N_2_O fluxes were not observed from the N richest organic amendment (digestate), but from the combinations of sewage sludge with compost, followed by the CC residue mixtures (Table 2). Hence, the N_2_O emission is not only depending on the N-content of the organic amendments, but also in which form the N-source is provided to the microorganisms. These observations are similar to our findings of the relatively weak correlation between C-content and CH_4_/CO_2_ fluxes. Additionally, we could not find any correlation of C/N or C-content to N_2_O fluxes (data not shown). Contrary to a recent study we also did not observe a linear relation between N fertilization and N_2_O emission (Shcherbak et al., 2014) in a study where all soil and environmental parameters were kept stable.

Only in case of the high organic amendment application we observed a second N_2_O flux peak after 21 d of incubation. In these incubations, the existing input of fresh N through the organic amendments was probably already processed and either turned into gaseous N, microbial N, or remains in refractory form. The microbial biomass or refractory N may release ammonium by mineralization, but this may take more time explaining the temporal pattern observed. Another explanation maybe that the soil parameters changed and stimulated the production of N_2_O again (e.g., through more anoxic zones). The results of the abundance analyses from these samples (Supplementary Table 3) revealed a strong increase of fungi in these samples, which could be causing the observed N_2_O production in our incubations. Fungi are known for possessing denitrification genes to produce N_2_O, but as yet have not been demonstrated to harbor N_2_O-reductase gene (Takaya, 2002; Shoun et al., 2012). It was also shown that denitrifying fungi already prefer drier conditions than denitrifying Bacteria (Chen et al., 2015). Additionally, since a SM of 40% normally does not favor denitrification processes (Skiba et al., 2002; Bateman and Baggs, 2005), changes in soil structure or chemistry (e.g., pH, O_2_ availability, aggregate composition) could have occurred leading to “hotspots” of N_2_O production as proposed to be responsible for local, temporary high denitrification activity (Groffman et al., 2009).

The water content has a more pronounced influence on the N_2_O emission than on the CH_4_ and CO_2_ fluxes. At low SM almost no N_2_O emission was detected. Since high SM reduces O_2_ availability and gas diffusivity and therefore will favor denitrification (Skiba et al., 2002), it can be assumed that in our incubation denitrification processes are the main source of N_2_O production. It was already observed in other studies that an increasing SM led to an increase of N_2_O production by denitrification (peak above 65% water-filled pore space), since the optimal SM concentration for nitrification peaks at around 55–65% water-filled pore space (Bateman and Baggs, 2005; Vargas et al., 2014; Sanz-Cobena et al., 2016). Contrary to this, the high amount of CC residue mixtures showed a strong increase in N_2_O emission at a low SM (Figure 4) just after 15 d. Even more surprising was the uptake of N_2_O after 28 d for the powdered CC residue mixture. This can either be caused by the high concentrations of N_2_O stimulating N_2_O reducers, or by a change in the soil characteristics (e.g., pH, O_2_ availability). Growth of fungi, which occurred in the CC residue bottles after some days of incubation, could also increase production of N_2_O activating the N_2_O-reducing community in the soil. It was shown recently that through application of plant residues, hotspots of N_2_O emission can occur, by enhanced water absorption from the plant residues which will lead to reduced O_2_ concentrations in the surrounding (Kravchenko et al., 2017). Combined with mineralized N and fungal growth this could explain the N_2_O peak caused by CC residues. To our knowledge this is the first time that such a behavior of N_2_O emission/consumption was observed after applying crop residues to the soil. More studies that confirm these results need to be conducted in the future.

### Abundance of Microorganisms in Relation to GHG Fluxes and Organic Amendment Application

Microbial dynamics following application of organic amendments clearly offers scope for modulating functional groups involved in consumption of GHGs. In this light, the CC residues materials showed the best results, by increasing the abundance of the denitrifiers (*nosZ)*, methanotrophs (*pmoA)*, and nitrogen fixers (*nifH)* genes, while only moderately increasing the nitrifiers (AOB) and methanogens (*mcrA)*. This could be either through the introduction of microbes already present in the organic amendments or stimulation of growth from indigenous microorganisms harboring these genes. Here, the effect is highly related to the amount of organic amendment applied to the soil. Small amounts of organic amendments have only a minor effect on the different microbial groups, which is also in accordance with the distinct lower GHG flux measurements from these incubations. On the opposite site, organic amendments cannot only increase the gene copy numbers, but can also lead to a decrease of microbial groups (AOA) in comparison to an un-amended soil.

The overall bacteria and fungi abundance correlate quite well with the CO_2_ respiration rates (*R*^2^ = 0.942/*R*^2^ = 0.858, respectively). The strong increase, especially in the CC residue application in bacterial and fungal abundance, could mainly occur due to the high application rate of the CC residue in our experiment. Normally, around 4- to 6-fold lower amounts of CC residues are plowed under in the field after the winter (Marinari et al., 2015; Coombs et al., 2017). However, we observe also an increase in the fungal abundance at the low amount of applied CC residues, which is comparable to recent studies (Maul et al., 2014).

The differences in abundance of the different groups are highly influenced by the different organic amendments that are used. For example, the application with the fungi based compost has a great effect (7-fold increase) on the abundance of the methanotrophs, compared to the green cut compost material which (like the other organic amendments) had only a doubling effect on the abundance of methanotrophs. Like mentioned before, a stimulation of rare-earth metal-dependent methanotrophs, which harbor the *XOXF* dependent methanol dehydrogenase gene, in these samples could be a possible explanation (Gu and Semrau, 2017; Krause et al., 2017). However, in a previous study (Ho et al., 2015) USCα *pmoA* sequences, which are known to poses the XOXF enzyme and is capable of atmospheric CH_4_ oxidation, was not detected in soil samples from the same location. This would rather support the hypotheses that the increase in *pmoA* copies is due to the introduction of methanotrophs by the organic amendment.

In contrast to the methanotrophic community, we observe more distinct differences of the effect of organic amendments on the methanogenic abundance. Especially organic amendments (compost and digestate) that undergo a treatment in which anoxic habitats are formed to provide a perfect environment for methanogens (Hellmann et al., 1997; Alburquerque et al., 2012). Especially, CC residue amendment increased the ratio of methanotrophs to methanogens, which can harbor a positive effect on the ratio of CH_4_ consumption to CH_4_ production (Conrad, 2007).

In our soil the newly found *nosZ* clade II (Jones et al., 2013) is 10- to 100-fold more abundant than *nosZ* clade I. While clade I is mainly associated with soil type (clay), nutrient status, total organic carbon, organic matter or C:N ratio, it is unclear what the drivers for the abundance of clade II in soils are (Highton et al., 2016; Hallin et al., 2017). Our soil is a clay soil, which would be expected to show a higher correlation to *nosZ* clade I bacteria, but instead we see a clear preference of N_2_O-reducers with a *nosZ* clade II gene. We think that the differentiation between the two clades cannot be broken down to just one or two single soil characteristics. More knowledge about the ecology of *nosZ* clade II bacteria, which seem to be the major drivers for soil N_2_O sink capacity (Jones et al., 2014; Domeignoz-Horta et al., 2016a), is necessary. This knowledge may be used to design strategies to enrich agricultural soils either directly with *nosZ* clade II microorganisms or using amendments that are rich in these denitrifiers. In our study almost all organic amendments had a stimulating effect on the two *nosZ* clades. The rise in N_2_O production may have stimulated the N_2_O-reducers during the incubation (Hallin et al., 2017).

The archaeal 16s rRNA gene and archaeal *amoA* are the only two genes that are decreasing during the incubation. For archaea and especially the AOA inside the archaea kingdom it was already shown that they are more affected by rewetting stress compared to bacteria and AOB (Conrad et al., 2014; Thion and Prosser, 2014). The decrease in the archaeal *amoA* seem to be higher with the addition of either CC residues, digestate or sewage sludge to the soil (Figure 7). Potentially, the high N-content in these organic amendments, along with the high water level is known to favor denitrification processes (Skiba et al., 2002). Furthermore, it is believed that the addition of fertilizer normally lead to an increase in the AOB/AOA ratio (Wertz et al., 2012; Hartmann et al., 2013; Kastl et al., 2015), since it was shown that AOB grow faster after the addition of fertilizer, this may also true for our study. Even though a recent study showed that this effect is not occurring in every occasion by showing that AOA and AOB had changed in the same way during an incubation (Orellana et al., 2018).

It is not surprising that the treatments with CC residues harbored the highest abundance of N-fixing bacteria, since 1/3 of the CC residues mixtures we added were legumes (Sprent et al., 2017). N-fixers cannot directly be linked to a GHG production or consumption, but can have an indirect effect on N_2_O production by converting N_2_ to NH_4_ which then can be consumed by nitrifiers in the soil (Galloway et al., 1995).

## Conclusion

In our study we analyzed different organic amendments and their influence on the GWP as well as functional microbial groups which are involved in GHG transformations in an agricultural soil. Our results indicate that compost amendments perform best with respect to the soil GWP calculated from the three major GHGs (CH_4_, CO_2_, N_2_O) and have a similar GWP as the un-amended soil (Table 2). Combinations of sewage sludge and digestate with both composts have also moderate effects on the soil GWP and will provide higher nutrients supply for plants. Although CC residues had the least favorable GWP, it still harbors a great long-term benefit to reduce GHG emissions from agricultural soils in manipulating the microbial communities. The CC residue amendment increased microbial groups that are involved in the reduction of GHGs (N_2_O-reducers, methanotrophs) or keeping the producing microbial community stable (methanogens, nitrifiers) compared to other organic amendments and the un-amended soil. This could provide a better GWP in the long-term. The next step would be to study the effect of plants on the GWP and have a deeper investigation of the associated microbial communities that are involved in GHG consumption and perform a longer running long-term incubation experiment to verify the short-term results. Further well-aerated agricultural soils need to be investigated in their potential as a sink for CH_4_, especially in combination with organic fertilizers and the potential of rare earth metals in these organic amendments. Understanding the underlying mechanisms of how organic fertilizers influence and possibly decrease GHG would allow us to develop a strategy to reduce GHG emission from agricultural soils without affecting the plant yield.

## Author Contributions

KB designed the study, performed the laboratory experiment, performed all lab work (flux measurements, nucleic-acid extractions, qPCR analysis, analytical analyses), performed statistical analysis, evaluated the data, and wrote the manuscript. SD helped with the set-up of the laboratory experiment, evaluated the data, and wrote the manuscript. GK helped with collecting the organic amendments and wrote the manuscript. PB designed the study, evaluated the data, and wrote the manuscript.

### Conflict of Interest Statement

The authors declare that the research was conducted in the absence of any commercial or financial relationships that could be construed as a potential conflict of interest.
