# Supplementary material for: Organic Residue Amendments to Modulate Greenhouse Gas Emissions From Agricultural Soils
Source: Front Microbiol. 2018 Dec 7;9:3035. doi: 10.3389/fmicb.2018.03035 (PMC6292959; doi:10.3389/fmicb.2018.03035)
Supplement: Supplementary file 1 [file Data_Sheet_1.docx]

**Supplementary**

**Supplementary Tables**

Sup. Tab. 1. Primers and PCR conditions used to amplify fragments of functional marker genes *nosZI/II*, archaeal *amoA,* bacterial *amoA*, *nifH, pmoA*, archaeal and bacterial 16S rRNA genes as well as fungal 18S rRNA gene by qPCR.

| **Gene** | **Primer sets** | **Forward primer** | **Reverse primer** | **PCR conditions** | **PCR product length (bp)** | **References** |
| --- | --- | --- | --- | --- | --- | --- |
| *nosZI* | nosZ2F/  nosZ2R | CGCRACGGCAASAAGGTSMSSGT | CAKRTGCAKSGCRTGGCAGAA | 95 °C/5min, 45 cycles (95°C/10sec, 64°C/10sec, 72°C/20sec), 65 to 95°C (+0.5°C/sec) for denaturation curve. | 267 | (Henry et al., 2006) |
| *nosZII* | nosZ-II-F/  nosZ-II-R | CTIGGICCIYTKCAYAC | GCIGARCARAAITCBGTRC | 95 °C/15min, 40 cycles (95°C/15sec, 54°C/30sec, 72°C/30sec, 80°C/15sec), 65 to 95°C (+0.5°C/sec) for denaturation curve. | 745 | (Jones et al., 2013) |
| Archaeal *amoA* | Arch-amoAF/  Arch-amoAR | STAATGGTCTGGCTTAGACG | GCGGCCATCCATCTGTATGT | 95 °C/15min, 40 cycles (95°C/10sec, 64°C/10sec, 72°C/20sec), 65 to 95°C (+0.5°C/sec) for denaturation curve. | 635 | (Francis et al., 2005) |
| Bacterial *amoA* | amoA1F/  amoA2R | GGGGTTTCTACTGGTGGT | CCCCTCKGSAAAGCCTTCTTC | 95 °C/10min, 40 cycles (95°C/10sec, 65°C/25sec, 72°C/30sec), 65 to 95°C (+0.5°C/sec) for denaturation curve. | 491 | (Rotthauwe et al., 1997) |
| *nifH* | PolF/  PolR | TGCGAYCCSAARGCBGACTC | ATSGCCATCATYTCRCCGGA | 95 °C/2min, 40 cycles (95°C/5sec, 54°C/10sec, 72°C/20sec), 60 to 95°C (+1°C/sec) for denaturation curve. | 360 | (Poly et al., 2001) |
| *mcrA* | mlas/  mcrA-rev | GGTGGTGTMGGDTTCACMCARTA | CGTTCATBGCGTAGTTVGGRTAGT | 95 °C/3min, 40 cycles (95°C/10sec, 60°C/10sec, 72°C/25sec), 65 to 95°C (+1°C/sec) for denaturation curve. | 645 | (Steinberg and Regan, 2008) |
| *pmoA* | A189/  Mb661R | GGNGACTGGGACTTCTGG | CCGGMGCAACGTCYTTACC | 95 °C/3min, 45 cycles (95°C/10sec, 58°C/15sec, 72°C/25sec, 82°C/10sec), 70 to 99°C (+1°C/sec) for denaturation curve. | 472 | (Costello and Lidstrom, 1999) |
| Archaeal  16S rRNA gene | 915/  1017r | AGGAATTGGCGGGGGAGCAC | GGCCATGCACCWCCTCTC | 95 °C/10min, 40 cycles (95°C/10sec, 60°C/10sec, 72°C/20sec), 65 to 95°C (+0.5°C/sec) for denaturation curve. | 112 | (Klindworth et al., 2013) |
| Bacterial  16S rRNA gene | Eub338/ Eub518 | ACTCCTACGGGAGGCAGCAG | ATTACCGCGGCTGCTGG | 95 °C/3min, 40 cycles (95°C/10sec, 53°C/10sec, 72°C/25sec), 60 to 95°C (+1°C/sec) for denaturation curve. | 180 | (Fierer et al., 2005) |
| Fungal  18S rRNA gene | FF390/  FR1 | CGATAACGAACGAGACCT | AICCATTCAATCGGTAIT | 95 °C/2min, 40 cycles (95°C/5sec, 52°C/10sec, 72°C/20sec), 65 to 95°C (+0.5°C/sec) for denaturation curve. | 390 | (Vainio and Hantula, 2000) |

Sup. Tab. 2. Abundance analyses measured with qPCR of the overall bacterial, archaeal and fungal community as well as of several functional marker genes (*amoA*, *nosZ*, *nifH*, *pmoA*) that are involved in producing or reducing GHG in soil, under a soil moisture content of 65%. (n = 3 ± STD)

| **Treatment** | **Archaeal l6S rRNA** | **Bacteria1 16S rRNA** | **Fungal 18S rRNA** | **Archaeal *amoA*** | **Bacterial *amoA*** | ***nifH*** | ***nosZ clade I*** | ***nosZ clade II*** | ***mcrA*** | ***pmoA*** |
| --- | --- | --- | --- | --- | --- | --- | --- | --- | --- | --- |
| Initial soil | 2.5E+08  ± 5.1E+07 | 1.8E+09  ± 2.2E+08 | 5.5E+06  ± 9.0E+05 | 1.4E+08  ± 2.6E+07 | 1.4E+07  ± 3.4E+06 | 6.8E+06  ± 2.9E+06 | 1.0E+06  ± 2.2E+05 | 2.4E+07  ± 5.0E+06 | 1.2E+05  ± 1.9E+04 | 1.3E+06  ± 3.1E+05 |
| Un-amended | 1.5E+08  ± 4.7E+07 | 2.2E+09  ± 2.7E+08 | 4.3E+06  ± 1.3E+05 | 9.5E+07  ± 1.0E+07 | 8.1E+06  ± 1.2E+06 | 7.9E+06  ± 2.5E+05 | 9.4E+05  ± 2.1E+05 | 5.1E+07  ± 6.0E+06 | 1.3E+05  ± 1.9E+04 | 1.6E+06  ± 4.0E+05 |
| ***High concentrations (20 t/ha)*** | | | | | | | | | |  |
| Compost1 | 2.6E+08  ± 5.7E+07 | 4.6E+09  ± 6.1E+08 | 1.0E+07  ± 3.5E+06 | 1.0E+08  ± 1.4E+07 | 2.4E+07  ± 4.3E+06 | 9.5E+06  ± 2.5E+06 | 3.7E+06  ± 2.7E+05 | 1.4E+08  ± 2.5E+07 | 4.4E+05  ± 7.6E+04 | 2.8E+06  ± 4.1E+05 |
| Compost2 | 2.4E+08  ± 1.4E+07 | 3.0E+09  ± 3.1E+08 | 4.4E+06  ± 3.6E+05 | 1.3E+08  ± 1.2E+07 | 1.2E+07  ± 3.5E+06 | 9.2E+06  ± 1.9E+06 | 2.3E+06  ± 8.3E+05 | 4.4E+07  ± 5.5E+06 | 1.5E+06  ± 4.6E+05 | 6.5E+06  ± 2.0E+06 |
| Cut cover crop | 1.6E+08  ± 1.8E+07 | 1.3E+10  ± 5.7E+09 | 7.9E+07  ± 3.6E+07 | 5.4E+07  ± 5.0E+06 | 8.0E+07  ± 1.0E+07 | 2.4E+07  ± 3.0E+06 | 3.0E+07  ± 2.0E+07 | 1.6E+08  ± 3.2E+07 | 1.3E+05  ± 1.8E+04 | 3.3E+06  ± 9.4E+05 |
| Powdered cover crop | 1.4E+08  ± 3.6E+07 | 7.0E+09  ± 1.8E+09 | 1.7E+07  ± 2.6E+06 | 4.9E+07  ± 1.0E+07 | 1.9E+07  ± 3.1E+06 | 4.2E+07  ± 1.9E+07 | 5.3E+06  ± 2.3E+06 | 1.2E+08  ± 1.7E+07 | 1.5E+05  ± 2.7E+04 | 2.9E+06  ± 5.3E+05 |
| Digestate | 2.5E+08  ± 4.7E+07 | 4.4E+09  ± 2.4E+08 | 8.3E+06  ± 3.5E+05 | 4.0E+07  ± 6.4E+06 | 4.5E+07  ± 1.4E+07 | 7.1E+06  ± 2.1E+06 | 4.7E+06  ± 1.1E+06 | 9.6E+07  ± 3.6E+07 | 1.1E+06  ± 9.9E+04 | 2.0E+06  ± 5.4E+05 |
| Digestate + compost1 | 3.3E+08  ± 4.1E+07 | 3.7E+09  ± 4.8E+08 | 6.7E+06  ± 4.1E+05 | 3.5E+07  ± 3.7E+06 | 3.1E+07  ± 4.8E+06 | 1.4E+07  ± 2.8E+06 | 3.2E+06  ± 8.4E+05 | 6.3E+07  ± 2.1E+07 | 1.9E+06  ± 1.1E+06 | 2.2E+06  ± 3.6E+05 |
| Digestate + compost2 | 3.5E+08  ± 1.1E+08 | 3.4E+09  ± 4.8E+08 | 5.6E+06  ± 1.6E+06 | 4.1E+07  ± 7.0E+06 | 2.5E+07  ± 4.3E+06 | 1.3E+07  ± 1.3E+06 | 2.6E+06  ± 7.4E+05 | 3.6E+07  ± 6.9E+06 | 1.7E+06  ± 6.7E+05 | 3.9E+06  ± 6.0E+05 |
| Sewage sludge + compost1 | 3.1E+08  ± 2.7E+07 | 4.0E+09  ± 1.2E+08 | 8.5E+06  ± 4.6E+05 | 2.9E+07  ± 5.6E+05 | 1.1E+08  ± 4.9E+07 | 7.6E+06  ± 2.9E+06 | 4.0E+06  ± 1.0E+06 | 1.3E+08  ± 3.1E+07 | 5.3E+05  ± 6.9E+04 | 2.1E+06  ± 2.9E+05 |
| Sewage sludge + compost2 | 3.3E+08  ± 9.6E+07 | 4.6E+09  ± 5.0E+08 | 8.4E+06  ± 2.5E+06 | 3.9E+07  ± 8.9E+06 | 8.0E+07  ± 5.5E+07 | 2.1E+07  ± 5.5E+06 | 7.3E+06  ± 1.9E+06 | 1.0E+08  ± 1.2E+07 | 1.5E+06  ± 4.8E+05 | 5.8E+06  ± 2.3E+05 |
| ***Low concentrations (5 t/ha)*** | | | | | | | | | |  |
| Compost1 | 1.2E+08  ± 7.0E+07 | 1.9E+09  ± 1.6E+08 | 6.2E+06  ± 2.4E+06 | 7.5E+07  ± 1.8E+07 | 1.1E+07  ± 1.9E+06 | 6.8E+06  ± 2.1E+06 | 1.4E+06  ± 2.9E+04 | 4.3E+07  ± 7.5E+06 | 1.5E+05  ± 3.7E+04 | 1.4E+06  ± 3.8E+05 |
| Compost2 | 1.1E+08  ± 7.4E+07 | 1.8E+09  ± 7.1E+07 | 5.6E+07  ± 3.0E+07 | 5.6E+07  ± 1.6E+07 | 9.9E+06  ± 1.5E+06 | 5.8E+06  ± 1.0E+06 | 1.1E+06  ± 2.0E+05 | 3.1E+07  ± 1.0E+07 | 2.4E+05  ± 5.0E+04 | 1.7E+06  ± 1.0E+05 |
| Cut cover crop | 1.3E+08  ± 1.3E+07 | 2.6E+09  ± 6.6E+08 | 1.7E+07  ± 5.2E+06 | 8.0E+07  ± 7.6E+06 | 1.4E+07  ± 2.2E+06 | 6.8E+06  ± 2.4E+06 | 1.9E+06  ± 3.2E+04 | 4.8E+07  ± 6.9E+06 | 1.2E+05  ± 2.2E+04 | 1.7E+06  ± 4.0E+05 |
| Powdered cover crop | 1.3E+08  ± 1.7E+07 | 2.4E+09  ± 5.9E+08 | 5.1E+06  ± 2.2E+06 | 6.2E+07  ± 1.9E+07 | 1.5E+07  ± 2.8E+06 | 9.5E+06  ± 7.8E+05 | 6.8E+05  ± 4.0E+05 | 2.9E+07  ± 1.6E+07 | 1.3E+05  ± 2.3E+04 | 1.8E+06  ± 5.4E+05 |
| Digestate | 1.0E+08  ± 6.9E+07 | 2.0E+09  ± 4.5E+08 | 2.6E+06  ± 4.0E+05 | 1.8E+07  ± 2.4E+06 | 8.2E+06  ± 1.0E+06 | 6.6E+06  ± 1.2E+06 | 2.0E+06  ± 4.7E+05 | 6.0E+07  ± 1.8E+07 | 4.4E+05  ± 1.5E+05 | 1.1E+06  ± 6.7E+04 |
| Digestate + compost1 | 2.4E+08  ± 2.1E+07 | 1.9E+09  ± 1.4E+08 | 3.5E+06  ± 6.9E+05 | 2.9E+07  ± 3.9E+06 | 1.1E+07  ± 1.2E+06 | 1.5E+07  ± 1.8E+06 | 2.4E+06  ± 4.7E+05 | 5.7E+07  ± 6.0E+06 | 2.3E+05  ± 2.7E+04 | 1.4E+06  ± 9.4E+04 |
| Digestate + compost2 | 1.8E+08  ± 1.3E+07 | 1.6E+09  ± 2.5E+08 | 2.7E+06  ± 6.9E+06 | 3.6E+07  ± 1.7E+06 | 1.3E+07  ± 2.5E+06 | 1.1E+07  ± 2.1E+06 | 1.2E+06  ± 1.0E+05 | 4.4E+07  ± 9.8E+06 | 2.1E+05  ± 5.5E+04 | 1.7E+06  ± 5.3E+05 |
| Sewage sludge + compost1 | 1.1E+08  ± 5.4E+07 | 1.4E+09  ± 2.0E+08 | 3.0E+06  ± 5.3E+05 | 3.2E+07  ± 1.3E+06 | 2.3E+07  ± 5.1E+06 | 9.6E+06  ± 9.0E+05 | 1.6E+06  ± 2.9E+05 | 3.8E+07  ± 3.7E+06 | 1.7E+05  ± 3.5E+04 | 1.3E+06  ± 2.9E+04 |
| Sewage sludge + compost2 | 9.4E+07  ± 1.2E+07 | 1.4E+09  ± 6.2E+07 | 3.9E+06  ± 8.3E+05 | 1.8E+07  ± 4.3E+06 | 1.1E+07  ± 1.8E+07 | 1.0E+07  ± 3.7E+06 | 1.8E+06  ± 1.7E+05 | 3.6E+07  ± 7.2E+06 | 2.8E+05  ± 5.5E+04 | 1.7E+06  ± 3.6E+05 |

Sup. Tab. 3. Abundance analyses measured with qPCR of the overall bacterial, archaeal and fungal community as well as of several functional marker genes (*amoA*, *nosZ*, *nifH*, *mcrA*, *pmoA*) that are involved in producing or reducing GHG in soil, under a soil moisture content of 40% and high concentration (20 t/ha) of the OA cover crop. (n = 3 ± STD)

| **Treatment** | **Archaeal l6S rRNA** | **Bacteria1 16S rRNA** | **Fungal 18S rRNA** | **Archaeal *amoA*** | **Bacterial *amoA*** | ***nifH*** | ***nosZ clade I*** | ***nosZ clade II*** | ***mcrA*** | ***pmoA*** |
| --- | --- | --- | --- | --- | --- | --- | --- | --- | --- | --- |
| Cut cover crop | 2.2E+08  ± 3.1E+07 | 4.9E+09  ± 2.0E+08 | 2.8E+07  ± 2.6E+06 | 8.8E+07  ± 1.7E+07 | 2.9E+07  ± 4.7E+06 | 8.4E+06  ± 3.7E+06 | 5.3E+06  ± 1.4E+06 | 7.5E+07  ± 1.7E+07 | 1.5E+05  ± 3.5E+04 | 2.5E+06  ± 7.2E+05 |
| Powdered cover crop | 1.3E+08  ± 2.8E+07 | 3.2E+09  ± 5.6E+08 | 2.0E+07  ± 3.2E+06 | 9.3E+07  ± 2.2E+07 | 1.3E+07  ± 2.2E+06 | 1.0E+07  ± 3.7E+06 | 2.1E+06  ± 6.4E+05 | 6.2E+07  ± 1.7E+07 | 1.3E+05  ± 3.1E+04 | 1.6E+06  ± 6.0E+05 |

Sup. Tab. 4. Ratio of the abundance, measured with qPCR, of the initial microbial soil community and the microbial community after a 28d incubation based on the overall bacterial, archaeal and fungal community as well as of several functional marker genes (*amoA*, *nosZ*, *nifH*, *mcrA*, *pmoA*) that are involved in producing or reducing GHG in soil, under a soil moisture content of 40% and high concentration (20 t/ha) of the OA cover crop. Different letters indicate significant differences in the ratio of the individual genes (ANOVA: P value < 0.05). (n = 3 ± STD)

| **Treatment** | **Archaeal l6S rRNA** | **Bacteria1 16S rRNA** | **Fungal 18S rRNA** | **Archaeal *amoA*** | **Bacterial *amoA*** | ***nifH*** | ***nosZ clade I*** | ***nosZ clade II*** | ***mcrA*** | ***pmoA*** |
| --- | --- | --- | --- | --- | --- | --- | --- | --- | --- | --- |
| Cut cover crop | 0.892 a  ± 0.122 | 2.744 a  ± 0.334 | 4.995 a  ± 0.574 | 0.615 a  ± 0.153 | 2.092 a  ± 0.473 | 1.249 a  ± 0.261 | 5.102 a  ± 0.908 | 3.131 a  ± 0.462 | 1.268 a  ± 0.114 | 1.826 a  ± 0.263 |
| Powdered cover crop | 0.537 a  ± 0.112 | 1.764 b  ± 0.075 | 3.544 a  ± 0.666 | 0.647 a  ± 0.178 | 0.965 a  ± 0.217 | 1.565 a  ± 0.240 | 2.048 b  ± 0.473 | 2.582 a  ± 0.650 | 1.102 a  ± 0.084 | 1.058 a  ± 0.267 |

**Supplementary Figures**


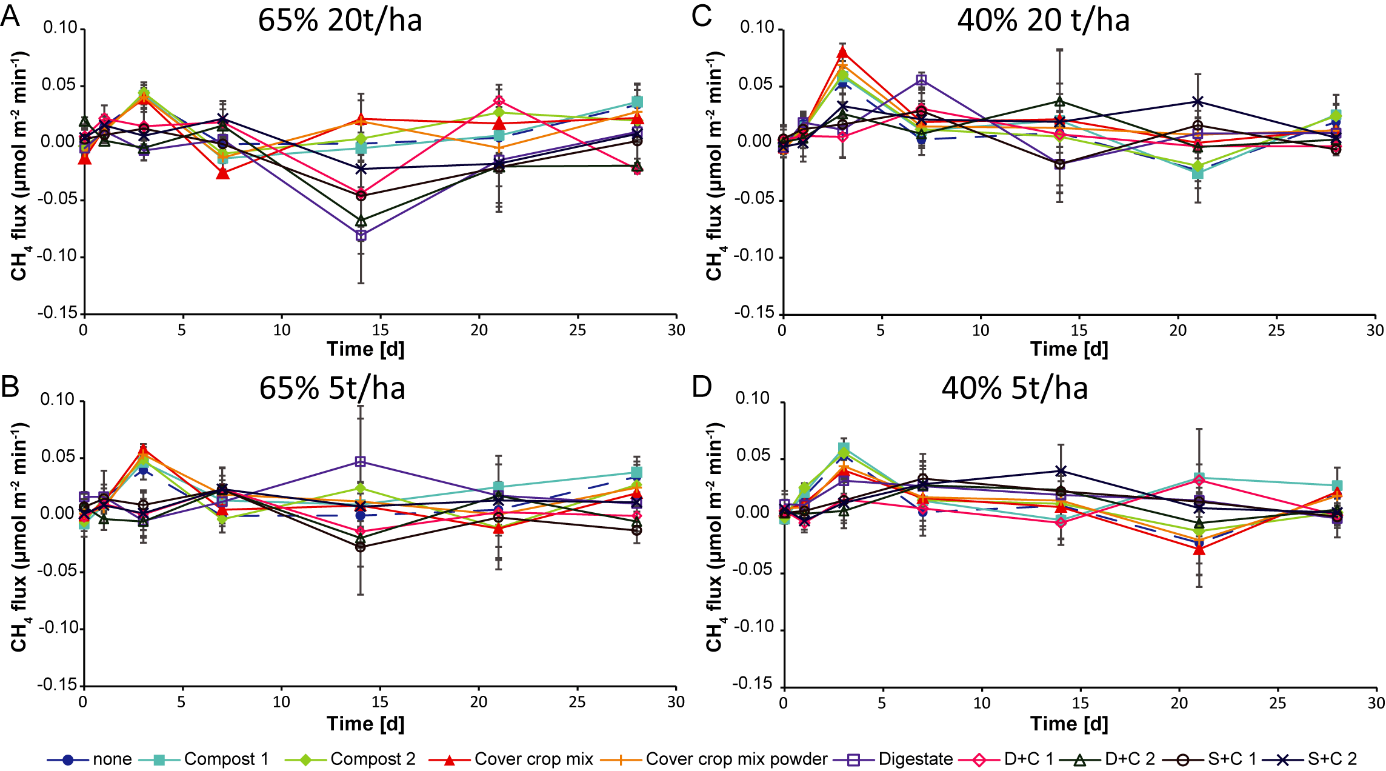


**Sup. Fig. 1.** Methane flux over the period of 28d in un-amended clay soil and after amendments with compost1, compost2, cover crop mixtures, digestate, digestate+compost1, digestate+compost2, sewage sludge+compost1 and sewage sludge+compost2 (mean ± SD; n = 3) at (A) high amount (20 t/ha) and high water content (65%), (B) low amount (5 t/ha) and high water content, (C) high amount and low water content (40%) and (D) low amount and low water content. Methane flux measurements were performed under ambient gas concentrations.


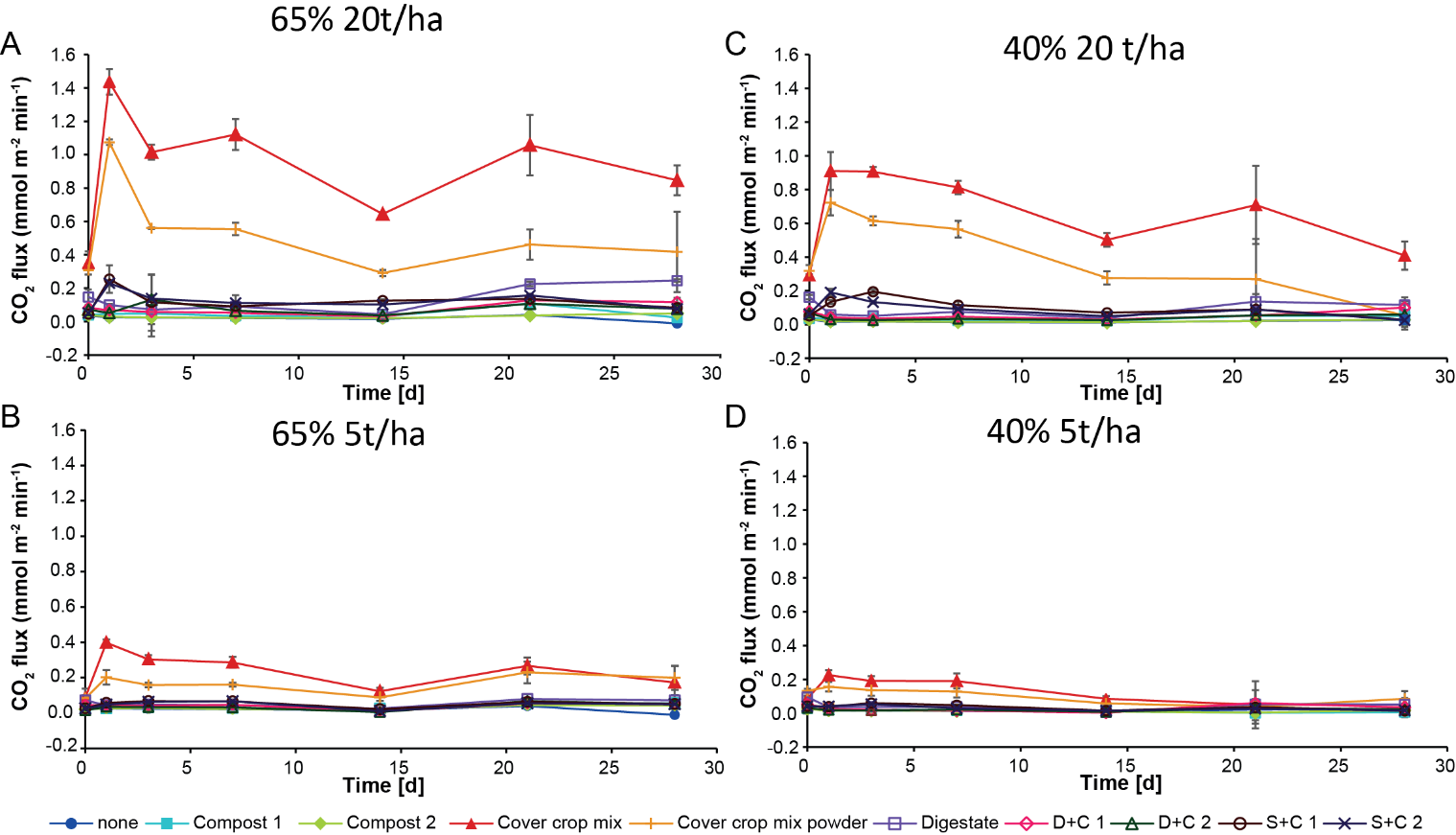


Sup. Fig. 2. Carbon dioxide flux over the period of 28d in un-amended clay soil and after amendments with compost1, compost2, cover crop mixtures, digestate, digestate+compost1, digestate+compost2, sewage sludge+compost1 and sewage sludge+compost2 (mean ± SD; n = 3) at (A) high amount (20 t/ha) and high water content (65%), (B) low amount (5 t/ha) and high water content, (C) high amount and low water content (40%) and (D) low amount and low water content. Carbon dioxide flux measurements were performed under ambient gas concentrations.


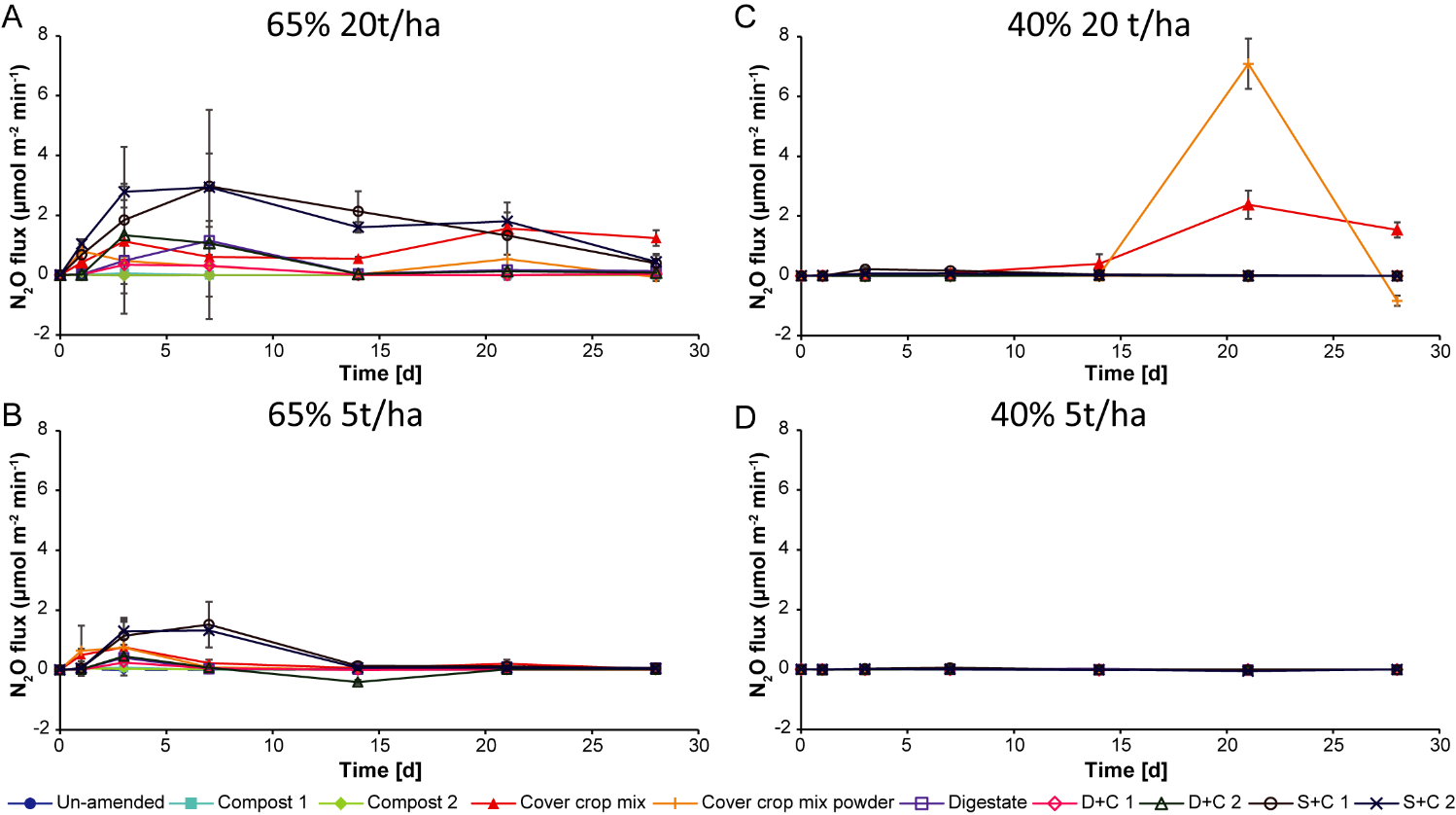


Sup. Fig. 3. Nitrous oxide flux over the period of 28d in un-amended clay soil and after amendments with compost1, compost2, cover crop mixtures, digestate, digestate+compost1, digestate+compost2, sewage sludge+compost1 and sewage sludge+compost2 (mean ± SD; n = 3) at (A) high amount (20 t/ha) and high water content (65%), (B) low amount (5 t/ha) and high water content, (C) high amount and low water content (40%) and (D) low amount and low water content. Nitrous Oxide flux measurements were performed under ambient gas concentrations.

**
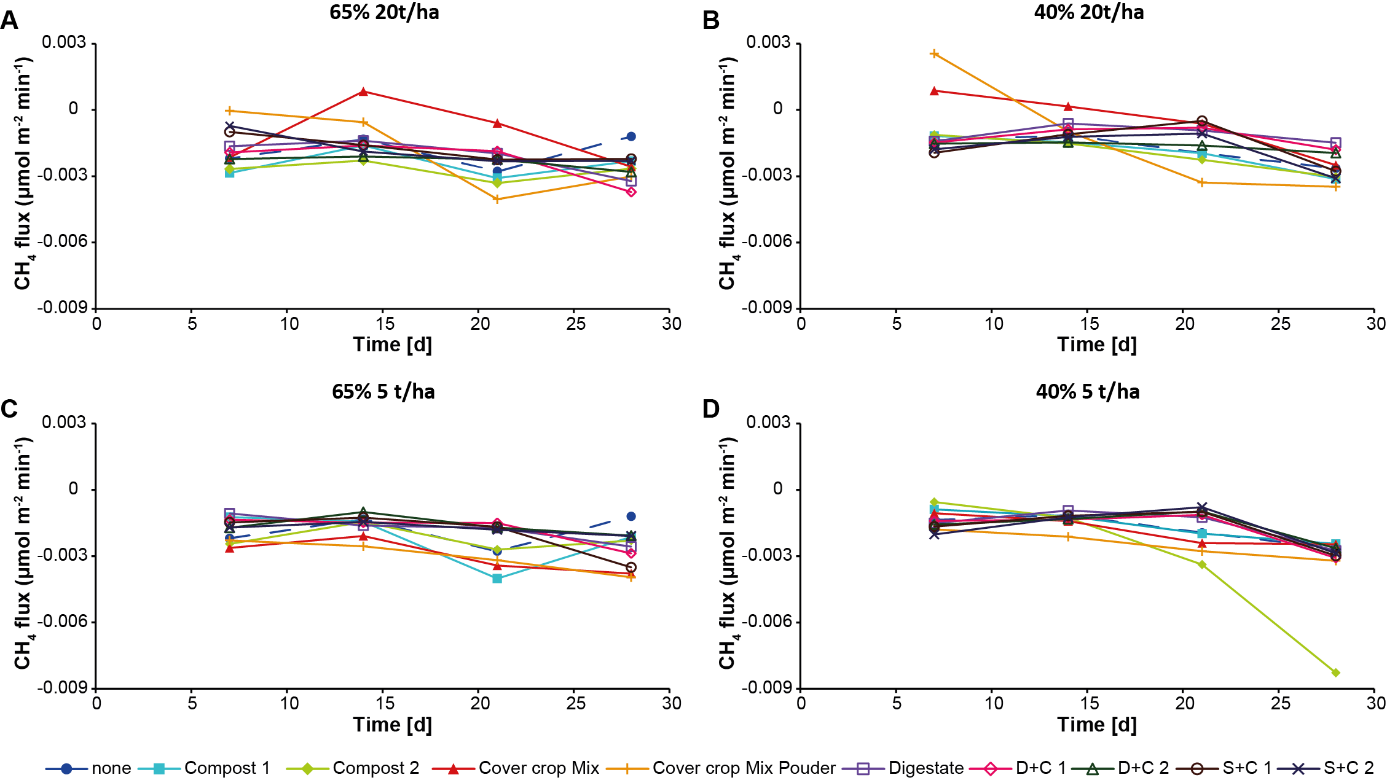
Sup. Fig. 4.** Methane uptake over the period of 28d in un-amended clay soil and after amendments with compost1, compost2, CC residues mixtures, digestate, digestate+compost1, digestate+compost2, sewage sludge+compost1 and sewage sludge+compost2 (mean ± SD; n = 3) at (A) high amount (20 t/ha) and high water content (65%), (B) low amount (5 t/ha) and high water content, (C) high amount and low water content (40%) and (D) low amount and low water content. Methane uptake were measured after the addition of 10 ppm CH_4_ at each sampling point.

**References**

Costello, A.M., and Lidstrom, M.E. (1999). Molecular characterization of functional and phylogenetic genes from natural populations of methanotrophs in lake sediments. *Applied and Environmental Microbiology* 65(11)**,** 5066-5074.

Fierer, N., Jackson, J.A., Vilgalys, R., and Jackson, R.B. (2005). Assessment of soil microbial community structure by use of taxon-specific quantitative PCR assays. *Applied and environmental microbiology* 71(7)**,** 4117-4120.

Francis, C.A., Roberts, K.J., Beman, J.M., Santoro, A.E., and Oakley, B.B. (2005). Ubiquity and diversity of ammonia-oxidizing archaea in water columns and sediments of the ocean. *Proceedings of the National Academy of Sciences of the United States of America* 102(41)**,** 14683-14688.

Henry, S., Bru, D., Stres, B., Hallet, S., and Philippot, L. (2006). Quantitative detection of the nosZ gene, encoding nitrous oxide reductase, and comparison of the abundances of 16S rRNA, narG, nirK, and nosZ genes in soils. *Applied and environmental microbiology* 72(8)**,** 5181-5189.

Jones, C.M., Graf, D.R., Bru, D., Philippot, L., and Hallin, S. (2013). The unaccounted yet abundant nitrous oxide-reducing microbial community: a potential nitrous oxide sink. *The ISME journal* 7(2)**,** 417.

Klindworth, A., Pruesse, E., Schweer, T., Peplies, J., Quast, C., Horn, M., et al. (2013). Evaluation of general 16S ribosomal RNA gene PCR primers for classical and next-generation sequencing-based diversity studies. *Nucleic acids research* 41(1)**,** e1-e1.

Poly, F., Monrozier, L.J., and Bally, R. (2001). Improvement in the RFLP procedure for studying the diversity of nifH genes in communities of nitrogen fixers in soil. *Research in Microbiology* 152(1)**,** 95-103.

Rotthauwe, J.-H., Witzel, K.-P., and Liesack, W. (1997). The ammonia monooxygenase structural gene amoA as a functional marker: molecular fine-scale analysis of natural ammonia-oxidizing populations. *Applied and environmental microbiology* 63(12)**,** 4704-4712.

Steinberg, L.M., and Regan, J.M. (2008). Phylogenetic comparison of the methanogenic communities from an acidic, oligotrophic fen and an anaerobic digester treating municipal wastewater sludge. *Applied and Environmental Microbiology* 74(21)**,** 6663-6671.

Vainio, E.J., and Hantula, J. (2000). Direct analysis of wood-inhabiting fungi using denaturing gradient gel electrophoresis of amplified ribosomal DNA. *Mycological research* 104(8)**,** 927-936.
